# Supplementary figures and images for: Combination and competition between path integration and landmark navigation in the estimation of heading direction
Source: PLoS Comput Biol. 2022 Feb 10;18(2):e1009222. doi: 10.1371/journal.pcbi.1009222 (PMC8865642; doi:10.1371/journal.pcbi.1009222)

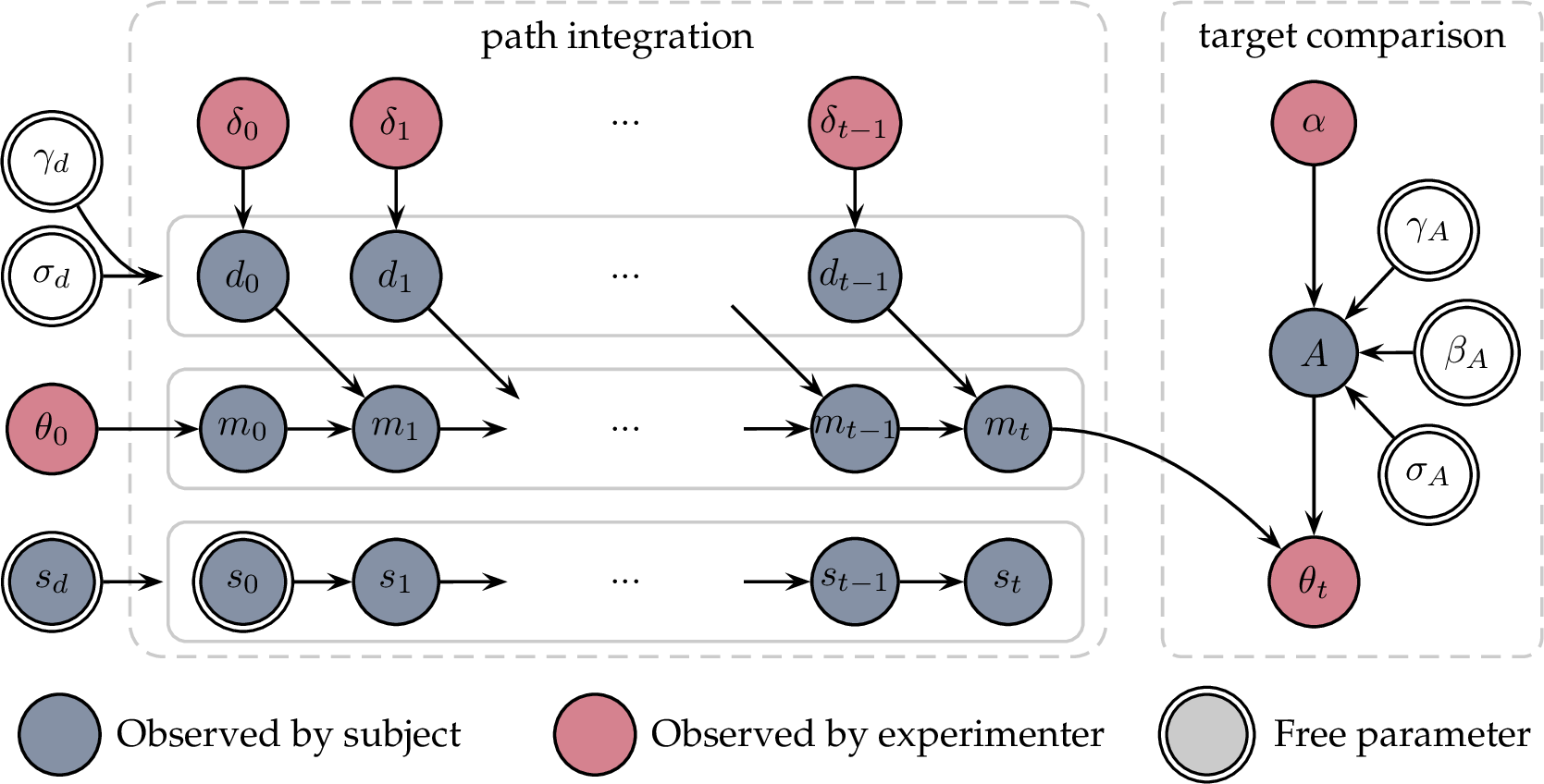

Supplement: S1 Fig — (TIF) [file pcbi.1009222.s001.tif]

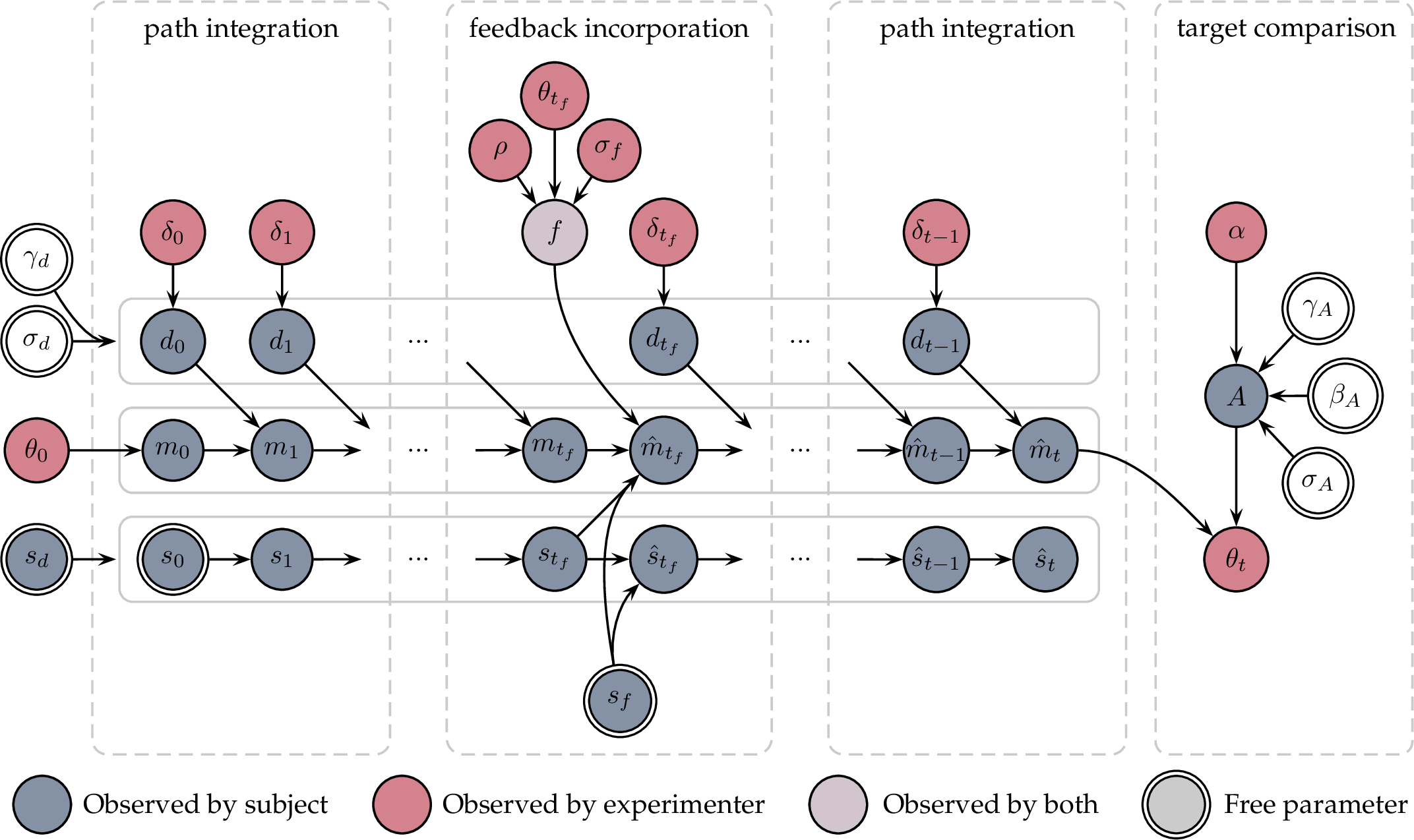

Supplement: S2 Fig — (TIF) [file pcbi.1009222.s002.tif]

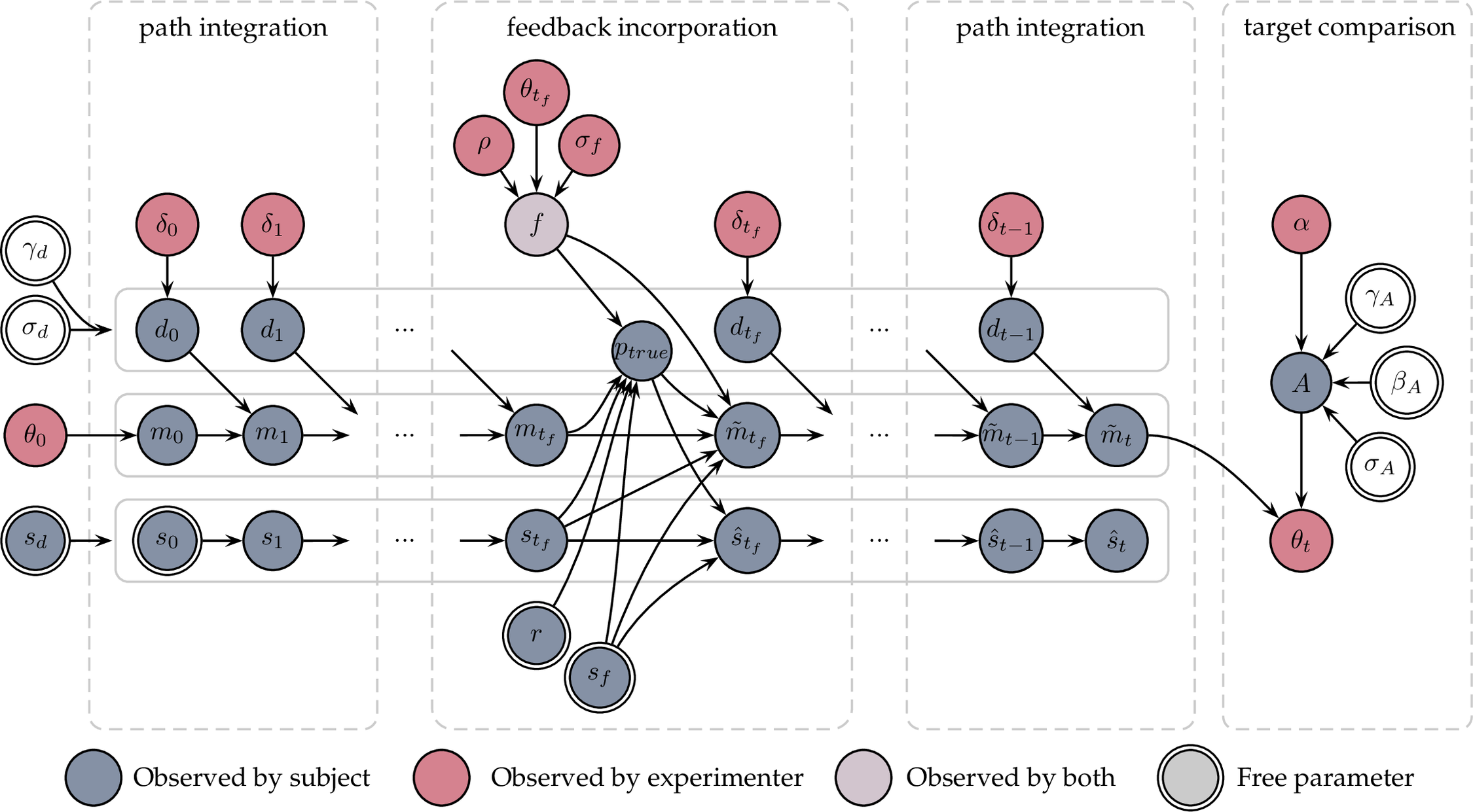

Supplement: S3 Fig — (TIF) [file pcbi.1009222.s003.tif]

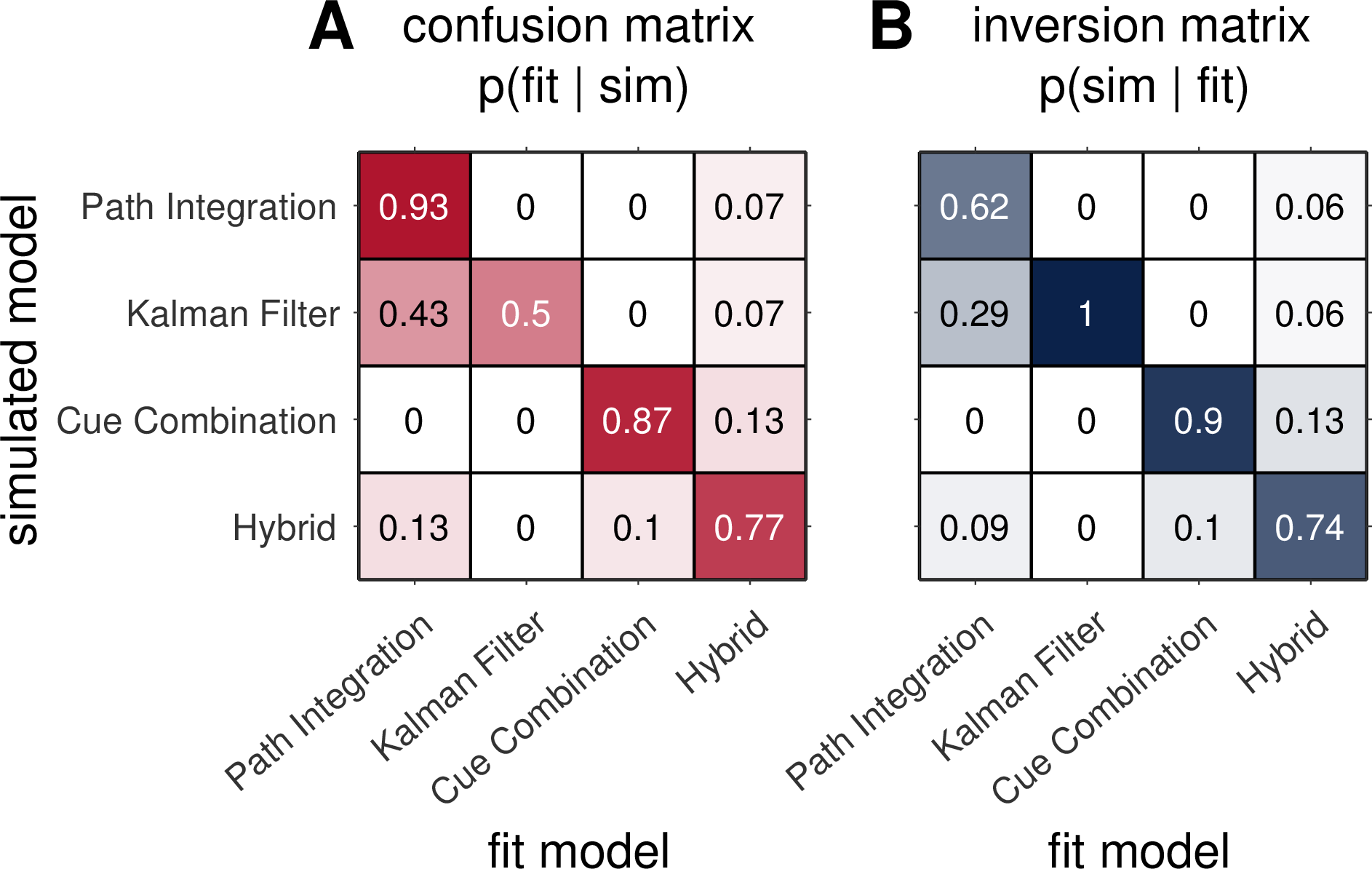

Supplement: S4 Fig — (TIF) [file pcbi.1009222.s004.tif]

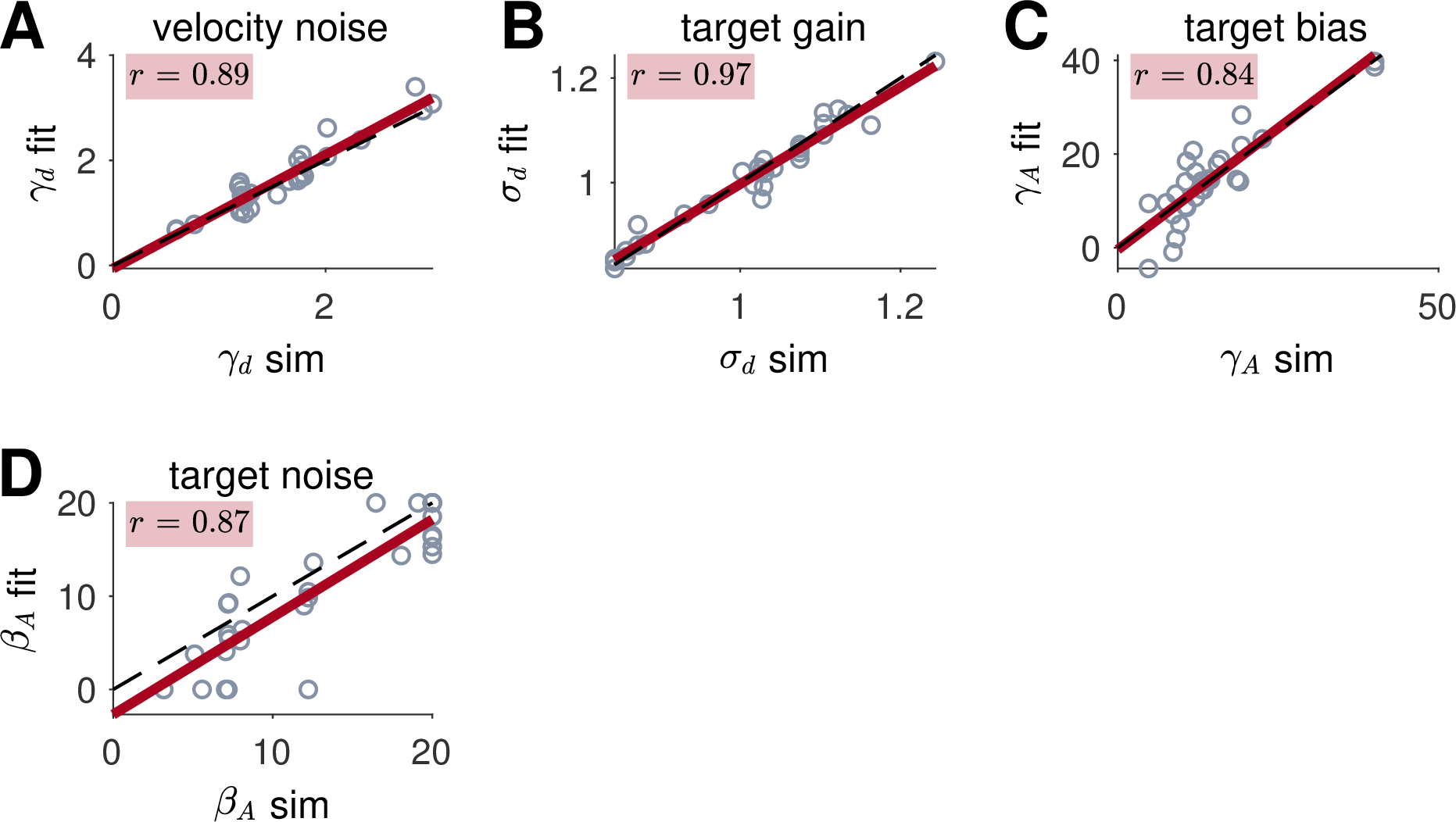

Supplement: S5 Fig — (TIF) [file pcbi.1009222.s005.tif]

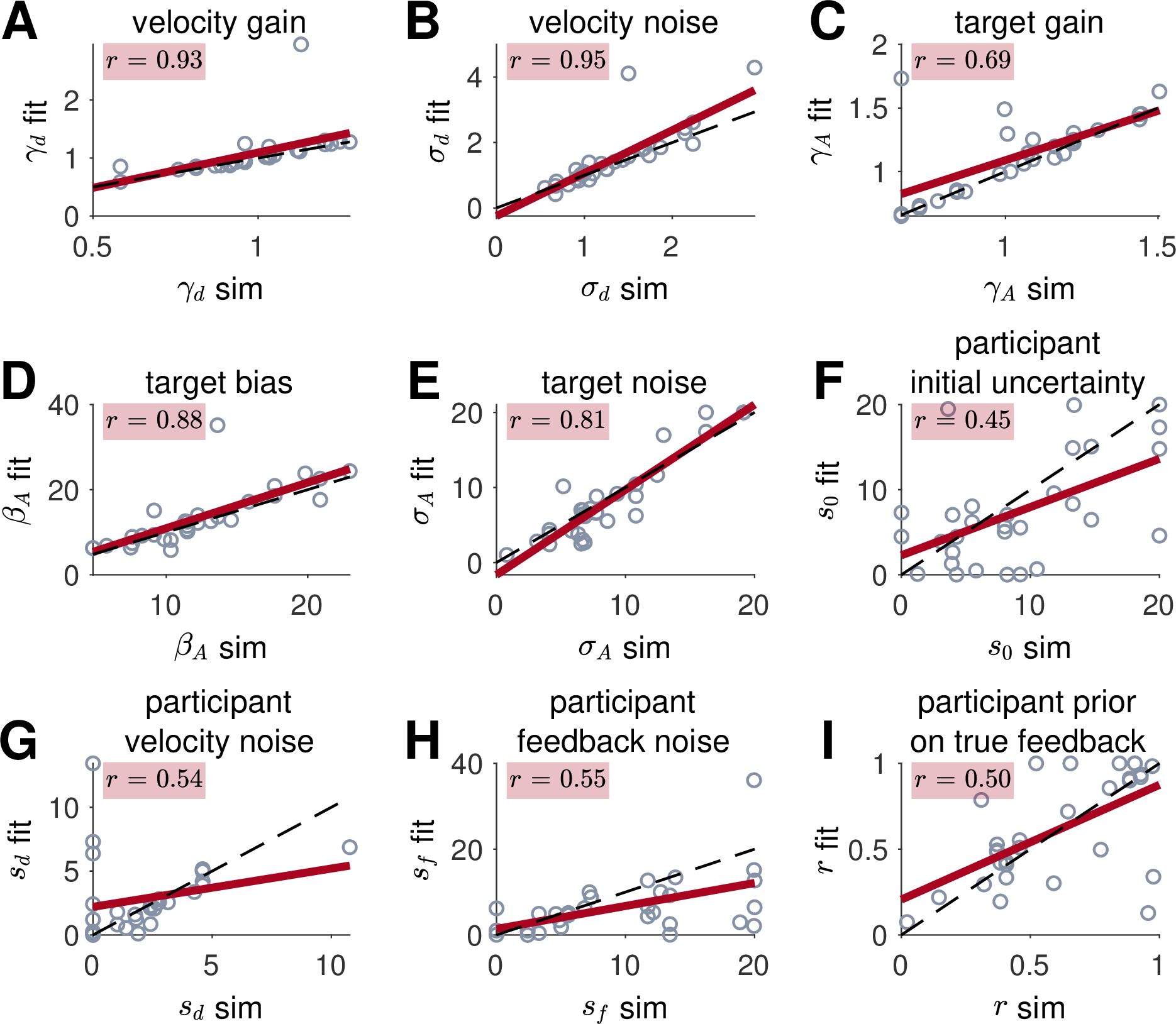

Supplement: S6 Fig — (TIF) [file pcbi.1009222.s006.tif]

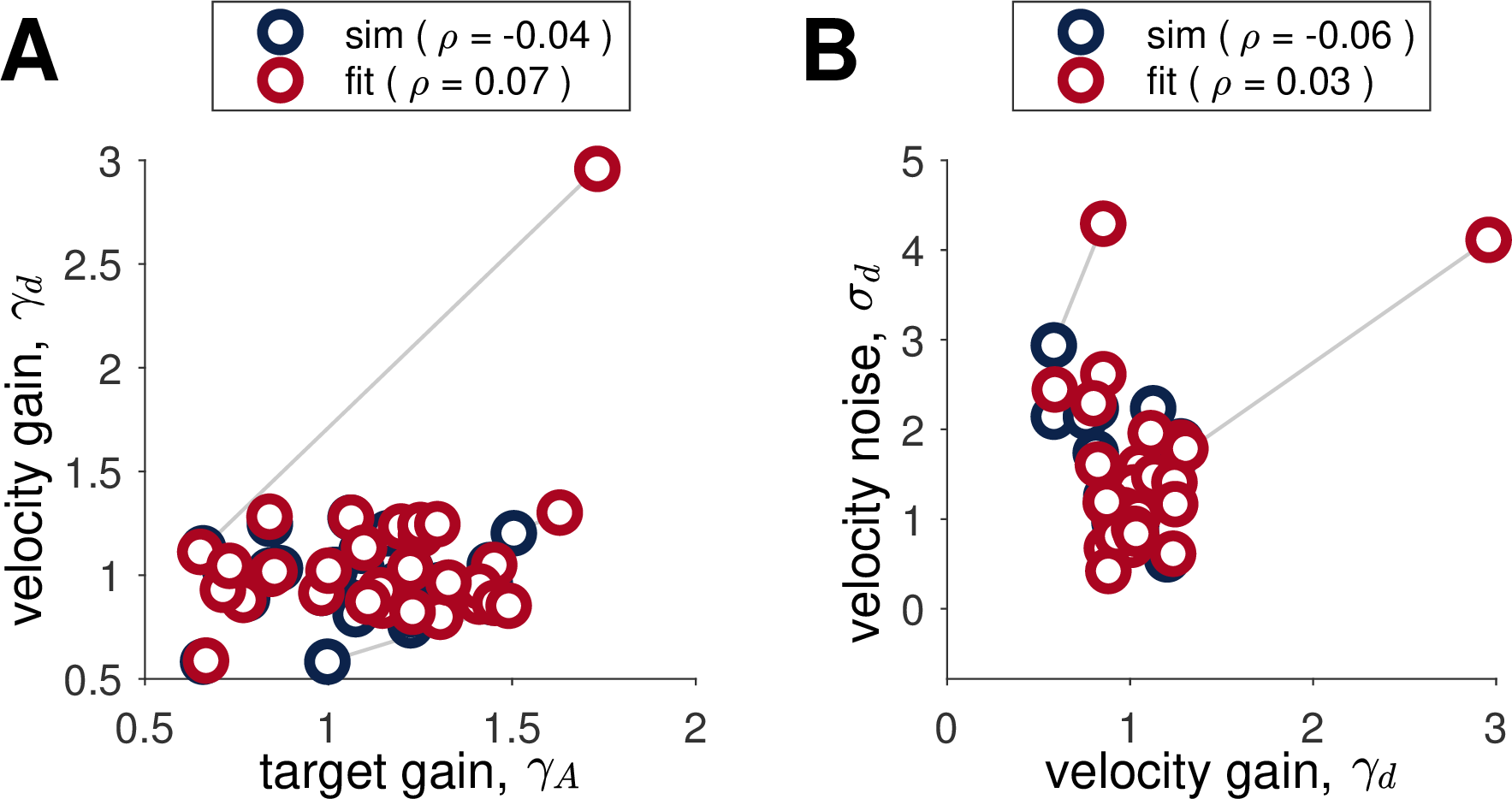

Supplement: S7 Fig — (TIF) [file pcbi.1009222.s007.tif]

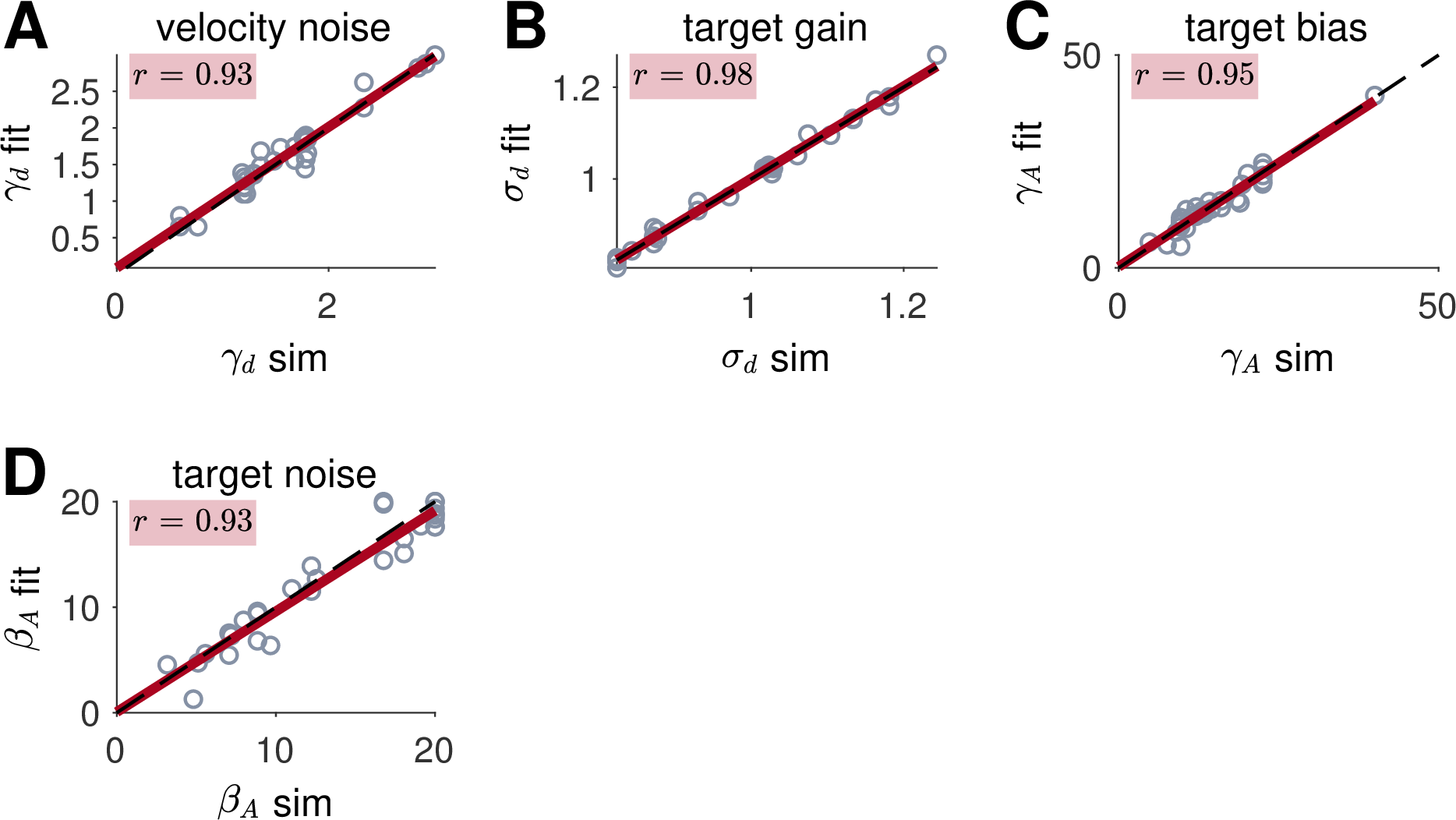

Supplement: S8 Fig — (TIF) [file pcbi.1009222.s008.tif]

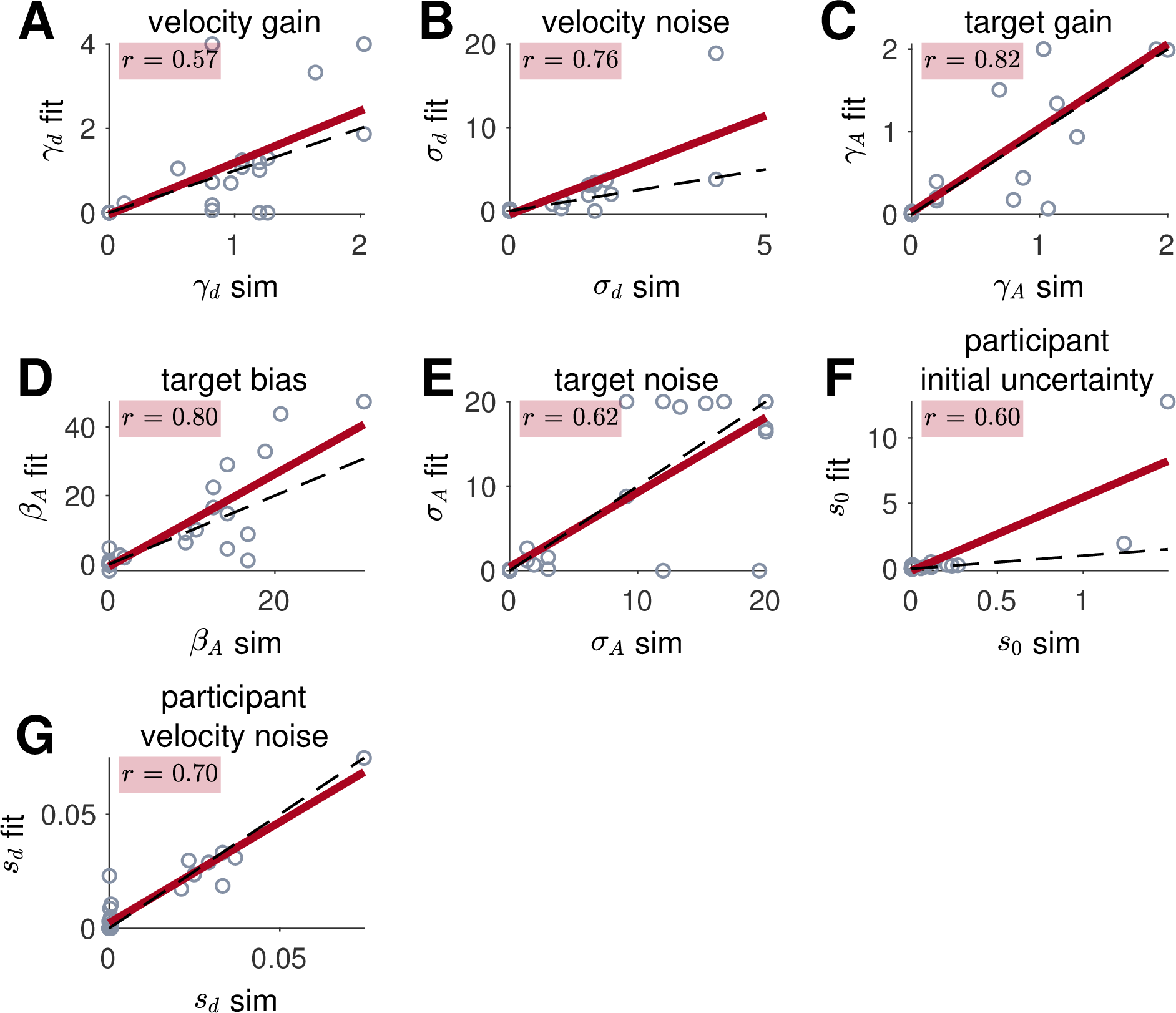

Supplement: S9 Fig — (TIF) [file pcbi.1009222.s009.tif]

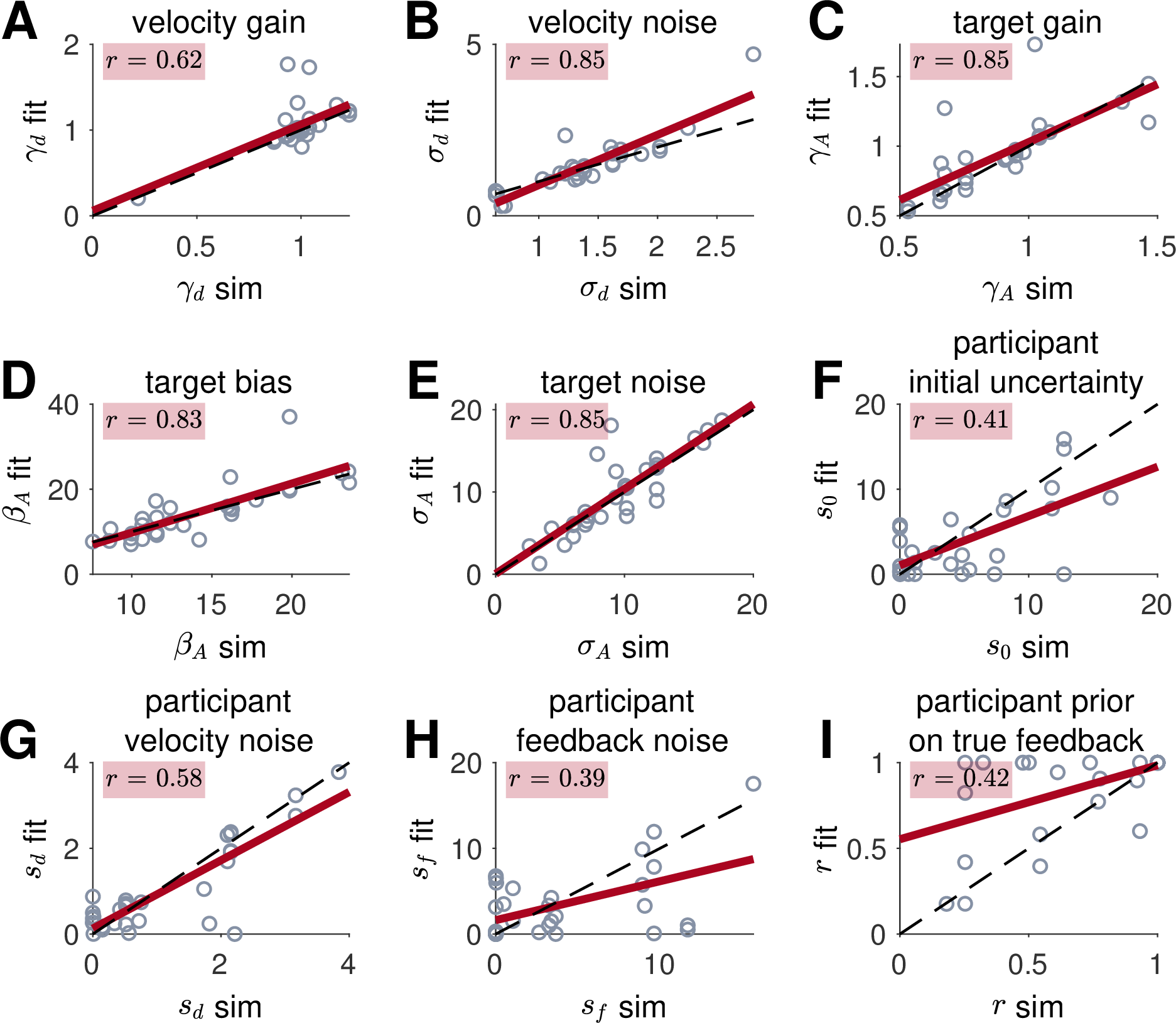

Supplement: S10 Fig — (TIF) [file pcbi.1009222.s010.tif]

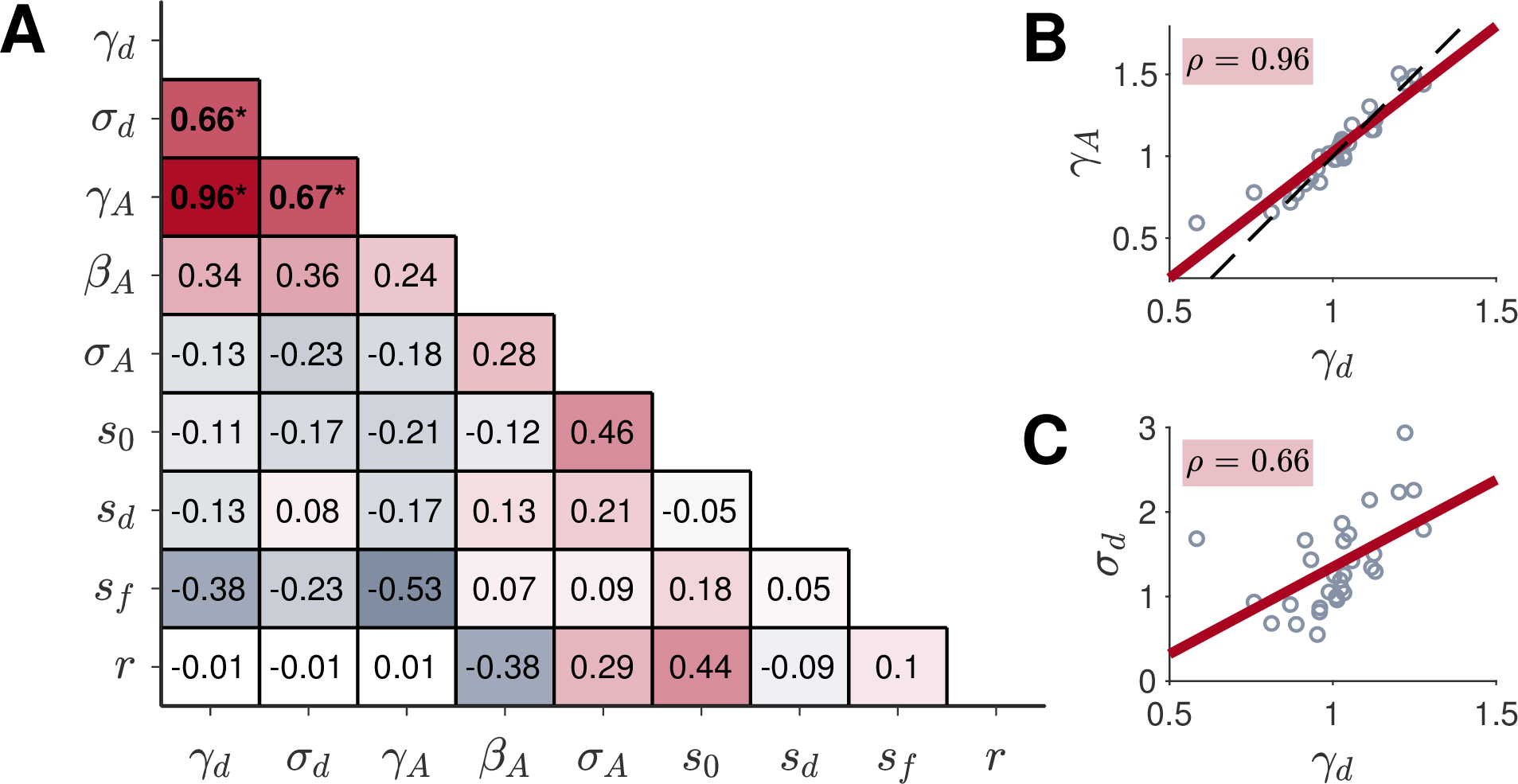

Supplement: S12 Fig — (TIF) [file pcbi.1009222.s012.tif]

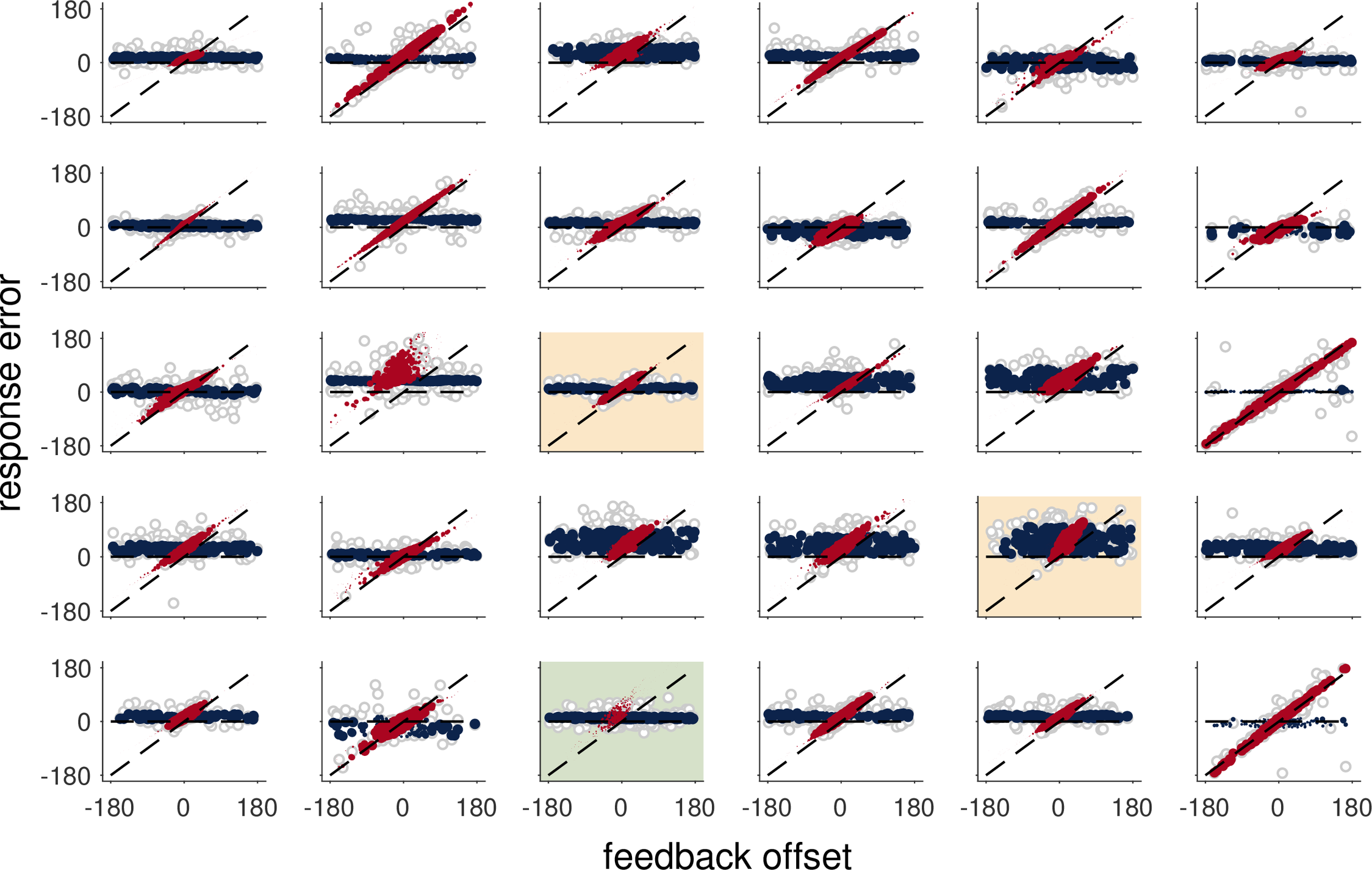

Supplement: S13 Fig — (TIF) [file pcbi.1009222.s013.tif]

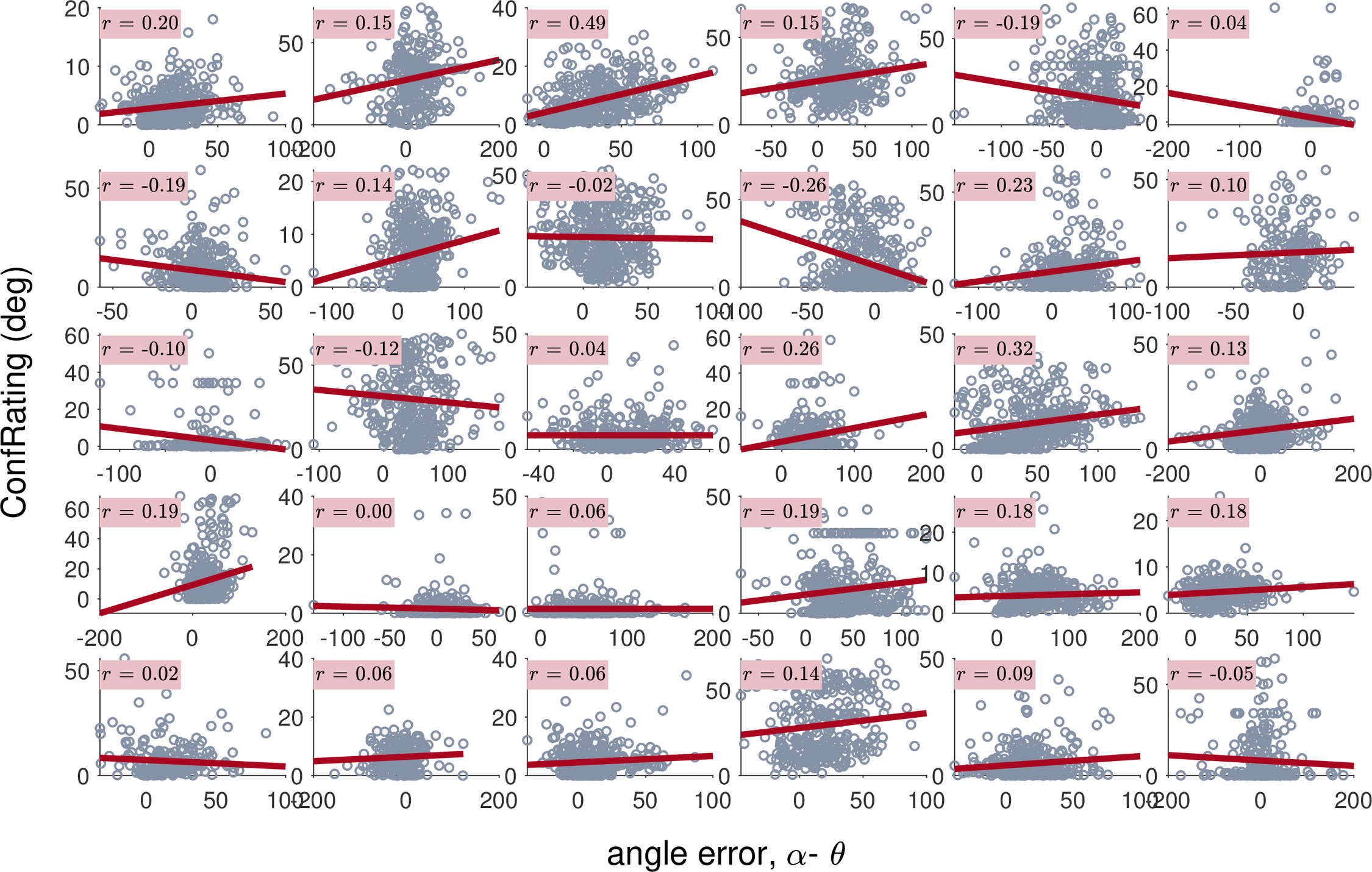

Supplement: S14 Fig — (TIF) [file pcbi.1009222.s014.tif]

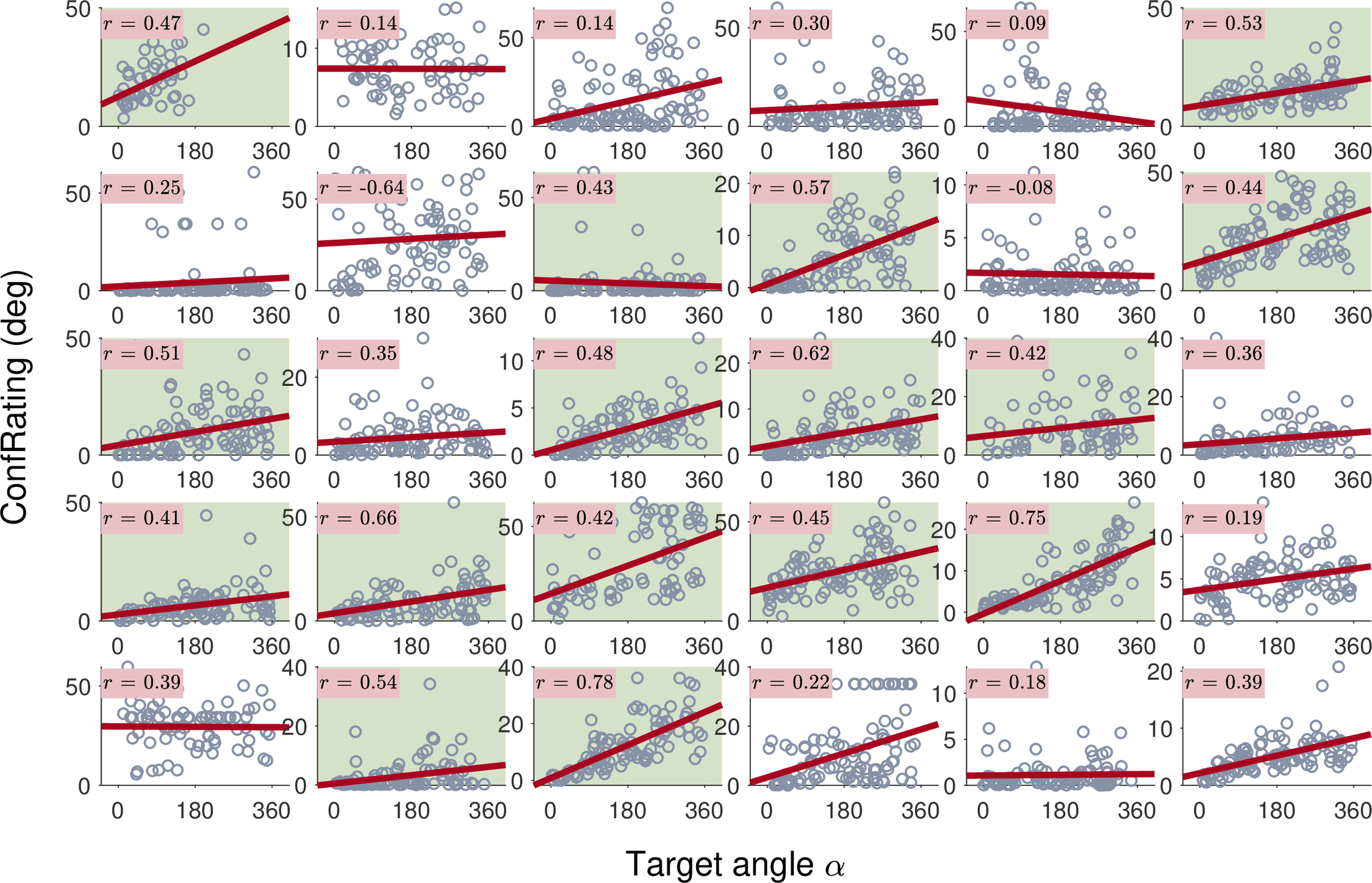

Supplement: S15 Fig — (TIF) [file pcbi.1009222.s015.tif]

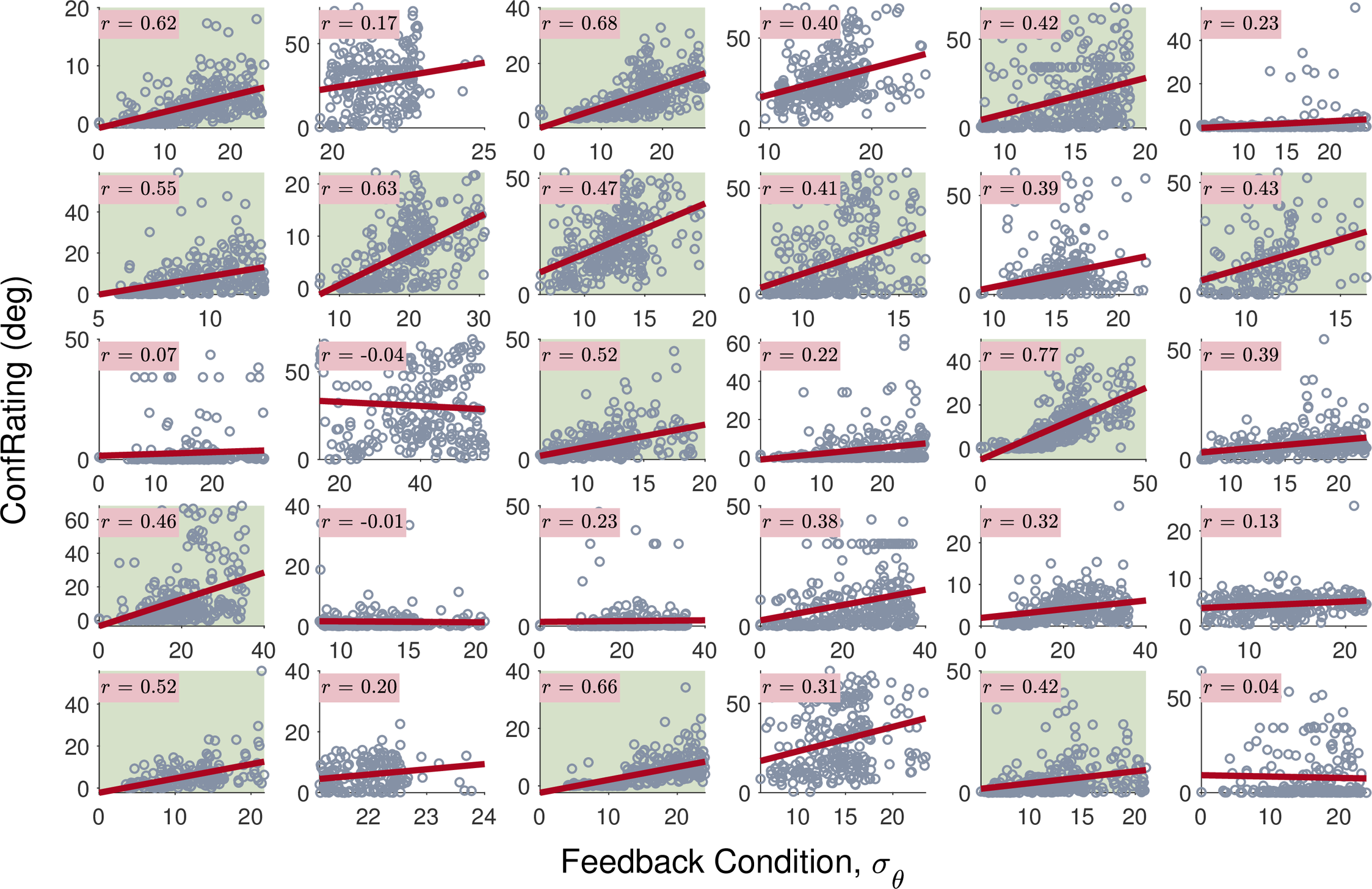

Supplement: S16 Fig — (TIF) [file pcbi.1009222.s016.tif]
