# Supplementary material for: Combination and competition between path integration and landmark navigation in the estimation of heading direction
Source: PLoS Comput Biol. 2022 Feb 10;18(2):e1009222. doi: 10.1371/journal.pcbi.1009222 (PMC8865642; doi:10.1371/journal.pcbi.1009222)
Supplement: S1 File — (PDF) [file pcbi.1009222.s017.pdf]

Supplementary Material: Combination and competition between path integration and landmark navigation in the estimation of heading direction

Contents

1 Computational Models **2**
- 1.1 Path Integration model 2
- 1.2 Kalman Filter model 5
- 1.3 Cue Combination model 9
- 1.4 Hybrid model 12

2 Bayesian decoding of target position **13**

3 Fitting simulated data **14**
- 3.1 Simulated data 14
- 3.2 Fitting simulated data 15
- 3.3 Model recovery 15
- 3.4 Parameter recovery 17

4 Parameter values for the Hybrid model **22**
- 4.1 Correlations between parameters 25

5 Model fit for all subjects **27**

6 Confidence Rating Correlations **28**

1 Computational Models

We built four models of the task which integrate the visual feedback in different ways. The simplest of these is the Path Integration model. This model ignores visual feedback completely and bases its estimate on pure path integration. As shown in the Results section, this model describes the behavior of at least one participant quite well and was used for all participants to fit data from the No Feedback condition.

The second model is the Kalman Filter model. This model integrates the visual feedback using the equations of the Kalman filter [1], which performs optimal cue combination under the assumption that the feedback error is Gaussian (which is not the case in our task because the feedback is sometimes sampled from a uniform distribution, see Eq. 1.

The third and fourth models extend the Kalman filter to better match the actual generative process of the experiment. These models assume that the feedback can be misleading and take this possibility into account by computing the probability that the feedback is 'true' (i.e. comes from the Gaussian distribution, $p(\text{true}|f)$) and false (i.e. comes from the uniform distribution, $p(\text{false}|f)$. The Cue Combination model, averages over this probability to form its estimate of heading. Conversely, the Hybrid model, samples from this probability, incorporating feedback just like the Kalman filter with probability $p(\text{true}|f)$ and ignoring the feedback with probability $p(\text{false}|f)$. In the following sections we develop each of these models in detail.

1.1 Path Integration model

We begin by modeling the case in which feedback is either absent (as in the No Feedback condition) or ignored (as in some participants). In this case, the estimate of heading is based entirely on path integration of vestibular cues. To make a response, i.e. to decide when to stop turning, we assume that participants compare their heading angle estimate, computed by path integration, with their memory of the target angle. Thus, the Path Integration model can be thought of as comprising two processes: a path integration process and a target comparison process (Fig S3).

Path integration In the encoding phase of the task, participants are guided through an initial turn of $-\alpha$ degrees to face heading angle, θ_0. In the retieval phase, they must then undo this rotation without visual feedback to return to $\theta_0 + \alpha$. For simplicity, and without loss of generality, we take the initial head direction on each trial to be $\theta_0 = 0^o$.

During retrieval, we assume that participants receive vestibular cues about their angular velocity as they rotate. For simplicity we model this process in discrete time, although the extension to continuous time is straightforward. We assume that on each time step t of the turn they receive a biased and noisy measure of their angular velocity, d_t, which is related

A) Graphical representation of the Path Integration model

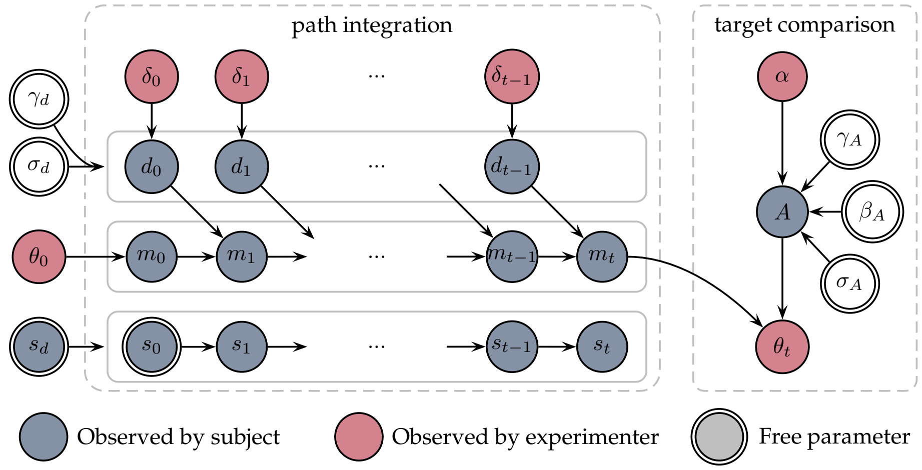

B) Mean and variance of error

$$\mathbb{E}\left[\theta_t - \alpha\right] = \frac{(\gamma_A - \gamma_d)\alpha + \beta_A}{\gamma_d} \quad ; \quad \mathbb{V}\left[\theta_t - \alpha\right] = \frac{\sigma_d^2 \alpha + \sigma_A^2}{\gamma_d^2}$$

Fig S1: The Path Integration model. (A) The model comprises two processes: path integration and target comparison. In the path integration process the models' estimate of heading angle, m_t (and corresponding uncertainty in this mean, s_t) is computed by integrating biased and noisy velocity information, d_t, over time. In the target comparison process, this estimate of heading is compared with the models' biased and noisy memory of the target angle, A, to decide when to stop at measured angle θ_t. Blue nodes correspond to variables that are 'observed' by the model (i.e. the participant) and can be used to compute the response. Red nodes correspond to variables that are observed by the experimenter and are the measurements we use to analyze behavior. White nodes correspond to parameters that are unobserved (by either the participant or the experimenter) describing imperfections in the coding of velocity (γ_d, σ_d) and target (γ_A, β_A, σ_A). Free parameters are denoted by a double line. To further distinguish between variables available to the participant and those that are not, we write variables available to the participant with Roman letters and variables that are not available to the participant with Greek letters. (B) The model predicts that both the mean and variance of the response error will be linear in target angle, α.

to their true angular velocity, δ_t by

$$d_t = \gamma_d \delta_t + \nu_t \tag{S1}$$

where γ_d denotes the gain on the velocity signal, which contributes to systematic under- or over-estimation of angular velocity. ν_t is zero-mean Gaussian noise with variance that increases in proportion to the magnitude of the angular velocity, $|\delta_t|$, representing a kind of Weber–Fechner law behavior [2],

$$\nu_t \sim \mathcal{N}\left(\nu_t | 0, \sigma_d^2 |\delta_t|\right) \tag{S2}$$

where σ_d^2 is a constant that determines the relationship between noise and angular velocity.

Next we assume that participants use this noisy velocity information to compute a probability distribution over their current heading angle.

$$p\left(\theta_t | d_{1:t-1}\right) = \mathcal{N}(\theta_t | m_t, s_t^2) \tag{S3}$$

where the mean of the distribution is given by

$$m_t = \sum_{i=1}^{t} d_t = \gamma_d \theta_t + \sum_{i=1}^{t} \nu_i = \gamma_d \theta_t + n_d \tag{S4}$$

where $n_d = \sum_{i=1}^{t} \nu_i$. The variance of the distribution is given by

$$s_t^2 = s_0^2 + \theta_t s_d^2 \tag{S5}$$

where s_0^2 is the participant's initial uncertainty in their location and s_d^2 is the participant's estimate of the variance of noise in their own vestibular system.

Strictly speaking, the behavior of the Path Integration model only depends on the mean, m_t, and we do not need to make the assumption that participants compute a full distribution over possible heading. However, as we shall see, computing both the mean and variance of $p\left(\theta_t | d_{1:t-1}\right)$ will be necessary for the models that incorporate feedback.

Target comparison Estimating the current heading angle is not enough to complete the task. In addition participants have to remember the target angle and compare it to their current estimate of heading. As with the encoding of velocity, we assume that this memory encoding is a noisy and biased process such that the participant's memory of the target angle is

$$A = \gamma_A \alpha + \beta_A + n_A \tag{S6}$$

where γ_A and β_A are the gain and bias on the memory that leads to systematic over- or under-estimation of the target angle, and n_A is zero mean Gaussian noise with variance σ_A^2.

In the Supplementary Section 2 we show how this form of a gain and bias on the target angle can result from Bayesian decoding of a noisy memory with no gain or bias.

To determine the response, we assume that participants stop moving when their current heading estimate matches the remembered angle. That is, when

$$m_t = A \tag{S7}$$

Substituting in the expressions for m_t and A, we get that the measured head angle when they stop, θ_t, will satisfy

$$\theta_t = \frac{1}{\gamma_d}\left(\gamma_A \alpha + \beta_A + n_A - n_d\right) \tag{S8}$$

Because the noise terms (n_A and n_d) are Gaussian, the measured error ($\theta_t - \alpha$) will also be Gaussian. Thus we can characterize the probability distribution over the measured error by its mean and variance

$$\mathbb{E}\left[\theta_t - \alpha\right] = \frac{(\gamma_A - \gamma_d)\alpha + \beta_A}{\gamma_d} \quad ; \quad \mathbb{V}\left[\theta_t - \alpha\right] = \frac{\sigma_d^2 \alpha + \sigma_A^2}{\gamma_d^2} \tag{S9}$$

The Path Integration model technically has seven free parameters (γ_d, σ_d, γ_A, β_A, σ_A, s_d, s_0). However, because the variance of the model's estimate does not affect θ_t, two of these parameters (s_d and s_0) cannot be estimated. In addition, the remaining five parameters all appear as ratios with γ_d giving us four free parameters in the Path Integration model. In practice when fitting the Path Integration model we set $\gamma_d = 1$ and interpret the remaining parameters as ratios (e.g. γ_A/γ_d, Table 2.

1.2 Kalman Filter model

Unlike the Path Integration model, which always ignores feedback, the Kalman Filter model always incorporates the visual feedback into its estimate of location. To model this process, we split the retrieval phase into four components: initial path integration, before the visual feedback is presented; feedback incorporation, when the feedback is presented; additional path integration, after the feedback is presented; and target comparison, to determine when to stop (Fig S2).

Initial path integration Initial path integration is identical to the Path Integration model. The model starts at a heading $\theta_0 = 0$ and integrates noisy angular velocity information over time to form an estimate of the mean, m_t and uncertainty, s_t, over the current heading angle θ_t.

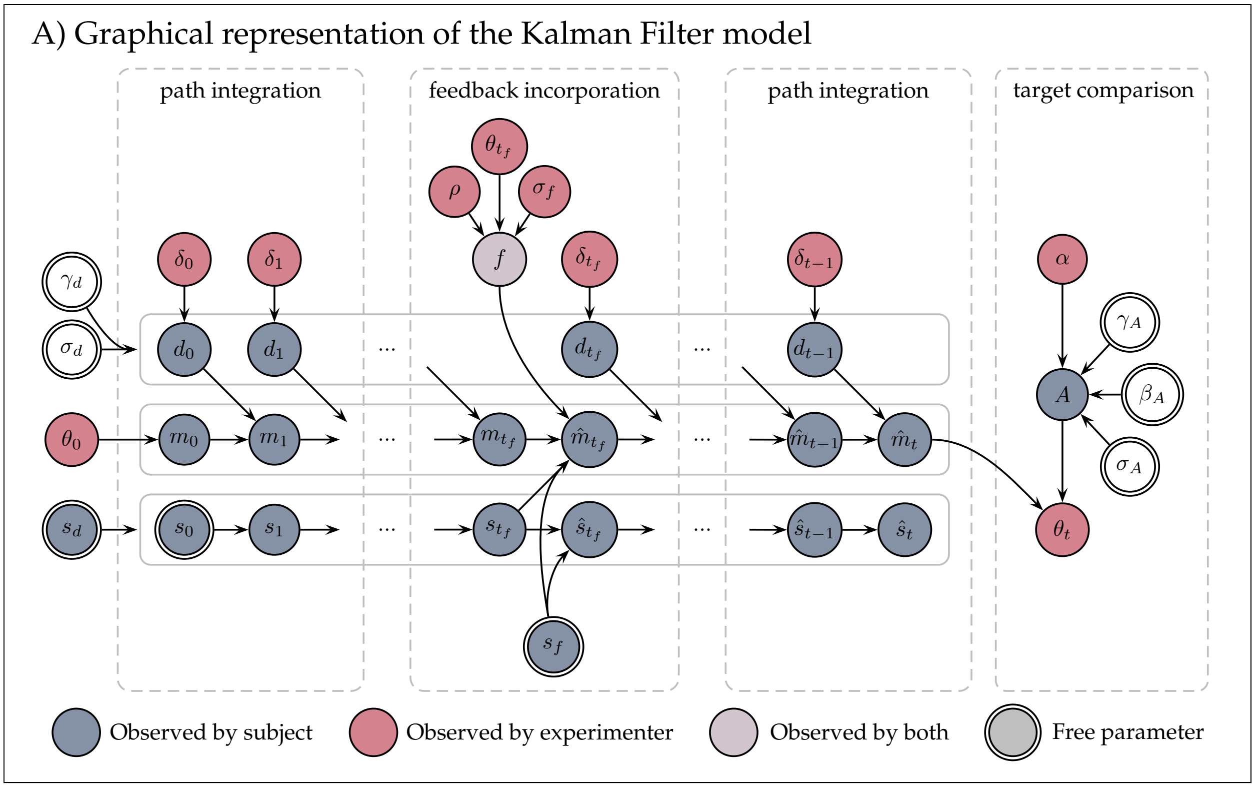

B) Mean and variance of error

$$\mathbb{E}\left[\theta_t - \alpha\right] = \frac{1}{\gamma_d}\left(\gamma_A \alpha - K_{t_f}(f - \gamma_d \theta_{t_f}) + b\right)$$
$$\mathbb{V}\left[\theta_t - \alpha\right] = \frac{1}{\gamma_d^2}\left(\sigma_A^2 + \left(\alpha - \left(2 - K_{t_f}\right) K_{t_f} \theta_{t_f}\right) \sigma_d^2\right)$$

Fig S2: Graphical representation of the Kalman Filter model. (A) The model comprises four processes: initial path integration, feedback incorporation, final path integration, and target comparison. The initial path integration process proceeds exactly as in the Path Integration model, estimating heading angle m_t from noisy velocity information d. In the feedback incorporation process, the path integration estimate is combined with the feedback f to form a combined estimate $\hat{m}_{t_f}$. In the final path integration process, the model incorporates the new noisy velocity information to update the combined estimate of heading. Finally, in the target comparison process, the combined estimate is compared with the remembered target angle A to generate the response θ_t. (B) Expressions for the mean and variance of the measured response error. Of note is that the mean is linear in both the target angle and the prediction error $f - \gamma_d \theta_{t_f}$.

Feedback incorporation At time t_f the feedback, f, is presented. The Kalman Filter model assumes that the feedback can be noisy, but that it is always carries some information about the true heading angle θ_{t_f}. In particular, this model assumes that the feedback angle is sampled from a Gaussian distribution centered on the true heading angle, θ_{t_f}, such that

$$p(f|\theta_{t_f}) = \mathcal{N}(f|\theta_{t_f}, s_f^2) \tag{S10}$$

where s_f^2 is the participant's estimate of the variance of the feedback.

With this Gaussian assumption for the likelihood of the feedback, the Kalman Filter model then combines the feedback with the estimate from path integration via Bayes rule

$$\begin{aligned} p(\theta_{t_f}|f, d_{1:t_f-1}) &\propto \overbrace{p(f|\theta_{t_f})}^{\text{feedback}} \quad \times \quad \overbrace{p(\theta_{t_f}|d_{1:t_f-1})}^{\text{path integration}} \\ &= \mathcal{N}(f|\theta_{t_f}, s_f^2) \times \mathcal{N}(\theta_{t_f}|m_{t_f}, s_{t_f}^2) \\ \implies p(\theta_{t_f}|f, d_{1:t_f-1}) &= \mathcal{N}(\theta_{t_f}|\hat{m}_{t_f}, \hat{s}_{t_f}^2) \end{aligned} \tag{S11}$$

where the mean and variance of this posterior distribution over (θ_{t_f}) are given by

$$\hat{m}_{t_f} = m_{t_f} + \frac{s_{t_f}^2}{s_{t_f}^2 + s_f^2}\left(f - m_{t_f}\right) \quad \text{and} \quad \hat{s}_{t_f}^2 = \frac{s_{t_f}^2 s_f^2}{s_{t_f}^2 + s_f^2} \tag{S12}$$

Note that the feedback updates the mean according to the prediction error $f - m_{t_f}$ weighted by the 'Kalman gain'

$$K_{t_f} = \frac{s_{t_f}^2}{s_{t_f}^2 + s_f^2} = \frac{s_0^2 + \theta_{t_f} s_d^2}{s_0^2 + \theta_{t_f} s_d^2 + s_f^2} \tag{S13}$$

The Kalman gain captures the influence of the prediction error on the estimate of heading angle. The more certain the model is that the feedback is accurate (i.e. smaller s_f) the closer the Kalman gain is to 1 and the larger the effect of the feedback. Conversely, the more certain the model is in its path integration estimate (i.e. smaller s_{t_f}) the closer the Kalman gain is to 0 and the smaller the effect of the feedback.

Additional path integration After the feedback has been incorporated, the model continues path integration using noisy velocity information. Thus the estimate of the mean continues to update as:

$$\hat{m}_t = \hat{m}_{t_f} + \sum_{i=t_f}^{t-1} \delta_i \tag{S14}$$

Substituting in the expressions for $\hat{m}_{t_f}$, d_i, and m_{t_f} we get

$$\begin{aligned}
\hat{m}_t &= m_{t_f} + K_{t_f}\left(f - m_{t_f}\right) + \sum_{i=t_f}^{t-1}\left(\gamma_d \delta_i + \nu_i\right) \\
&= \sum_{i=1}^{t_f-1}\left(\gamma_d \delta_i + \nu_i\right) + K_{t_f}\left(f - \sum_{i=1}^{t_f-1}\left(\gamma_d \delta_i + \nu_i\right)\right) + \sum_{i=t_f}^{t-1}\left(\gamma_d \delta_i + \nu_i\right) \\
&= \gamma_d \theta_t + K_{t_f}\left(f - \gamma_d \theta_{t_f}\right) + \underbrace{\sum_{i=1}^{t_f-1}(1 - K_{t_f})\nu_i + \sum_{i=t_f}^{t-1}\nu_i}_{\text{noise, } \epsilon}
\end{aligned} \tag{S15}$$

Target comparison Finally, the response is determined as the point at which $\hat{m}_t$ is equal to the noisy target angle, A

$$\hat{m}_t = \gamma_A \alpha + \beta_A + n_A \tag{S16}$$

Substituting in the expression for $\hat{m}_t$ and rearranging for the response angle gives

$$\theta_t = \frac{1}{\gamma_d}\left(\gamma_A \alpha - K_{t_f}\left(f - \gamma_d \theta_{t_f}\right) + \beta_A + n_A - \epsilon\right) \tag{S17}$$

This implies that the distribution of errors $(\theta_t - \alpha)$ is Gaussian with a mean given by

$$\mathbb{E}\left[\theta_t - \alpha\right] = \frac{1}{\gamma_d}\left(\gamma_A \alpha - K_{t_f}(f - \gamma_d \theta_{t_f}) + b\right) \tag{S18}$$

and a variance given by

$$\begin{aligned}
\mathbb{V}\left[\theta_t - \alpha\right] &= \frac{1}{\gamma_d^2}\left(\sigma_A^2 + (1 - K_{t_f})^2 \theta_{t_f} \sigma_d^2 + (\alpha - \theta_{t_f})\sigma_d^2\right) \\
&= \frac{1}{\gamma_d^2}\left(\sigma_A^2 + \left(\alpha - \left(2 - K_{t_f}\right) K_{t_f} \theta_{t_f}\right) \sigma_d^2\right)
\end{aligned} \tag{S19}$$

Note that, because s_f^2, s_0^2, and s_d^2 appear as part of a ratio in the equation for K_{t_f}, only two out of the three of these can be estimated from data. Thus, of the eight free parameters in the Kalman Filter model (γ_d, σ_d, γ_A, β_A, σ_A, s_0, s_d, and s_f), only seven can be estimated from the data. In practice, when fitting this model we set $s_f^2 = 1$ and interpret the participant's initial uncertainty and estimate of velocity noise as ratios, s_0/s_f and s_d/s_f respectively Table 2.

1.3 Cue Combination model

The Cue Combination model takes into account the possibility that the feedback will be misleading. To do this it computes a mixture distribution over heading angle with one component of the mixture assuming that the feedback is false and the other that the feedback is true. These two components are weighted according to the computed probability that the feedback is either false or true.

Mathematically, the Cue Combination model computes the probability distribution over heading angle by marginalizing over the truth of the feedback

$$p(\theta_{t_f}|f, d_{1:t_f}) = p(\theta_{t_f}|\text{false}, d_{1:t_f})p(\text{false}|f, d_{1:t_f}) + p(\theta_{t_f}|\text{true}, f, d_{1:t_f})p(\text{true}|f, d_{1:t_f}) \quad \text{(S20)}$$

where $p(\text{true}|f, d_{1:t_f}) = p_{true} = 1 - p_{false}$ is the probability that the feedback is true given the noisy velocity cues seen so far. Thus the Cue Combination model requires two steps to incorporate the feedback: first compute p_{true} and second average over p_{true} to determine how much to take the feedback into account.

Computing p_{true} Using Bayes rule, we can write the probability that the feedback is true, p_{true} as

$$p(\text{true}|f, d_{1:t_f}) = \frac{p(f|\text{true}, d_{1:t_f})p(\text{true})}{p(f|\text{true}, d_{1:t_f})p(\text{true}) + p(f|\text{false}, d_{1:t_f})p(\text{false})} \quad \text{(S21)}$$

where $p(f|\text{true}, d_{1:t_f})$ is the likelihood of the feedback assuming it is true, $p(f|\text{false}, d_{1:t_f})$ is the likelihood of the feedback assuming it is false, and $p(\text{true}) = 1 - p(\text{false})$ is the participant's estimate of the prior probability that the feedback is true.

The likelihood of the feedback given that it is true, $p(f|\text{true}, d_{1:t_f})$, can be computed by marginalizing over the estimate of heading direction as

$$p(f|\text{true}, d_{1:t_f}) = \int d\theta_{t_f} p(f|\theta_{t_f}, \text{true})p(\theta_{t_f}|d_{1:t_f}) \quad \text{(S22)}$$

where $p(\theta_{t_f}|d_{1:t_f})$ is the heading angle distribution computed by path integration and $p(f|\theta_{t_f}, \text{true})$ is the likelihood of the feedback given the heading angle, which we assume to be Gaussian; i.e.,

$$p(f|\theta_{t_f}, \text{true}) = \mathcal{N}(f|\theta_{t_f}, s_f^2) \quad \text{(S23)}$$

This implies that $p(f|\text{true}, d_{1:t_f})$ is the convolution of two Gaussians, which is itself another Gaussian

$$\begin{aligned} p(f|\text{true}, d_{1:t_f}) &= \mathcal{N}(f|\theta_{t_f}, s_f^2) \circledast \mathcal{N}(\theta_{t_f}|m_{t_f}, s_{t_f}^2) \\ &= \mathcal{N}(f|m_{t_f}, s_f^2 + s_{t_f}^2) \end{aligned} \quad \text{(S24)}$$

The likelihood of the feedback given that it is false, $p(f|\text{false}, d_{1:t_f})$, is simply a uniform distribution over f

$$p(f|\text{false}, d_{1:t_f}) = \mathcal{U}(f) = \frac{1}{2\pi} \quad \text{(S25)}$$

A) Graphical representation of the Cue Combination and Hybrid models

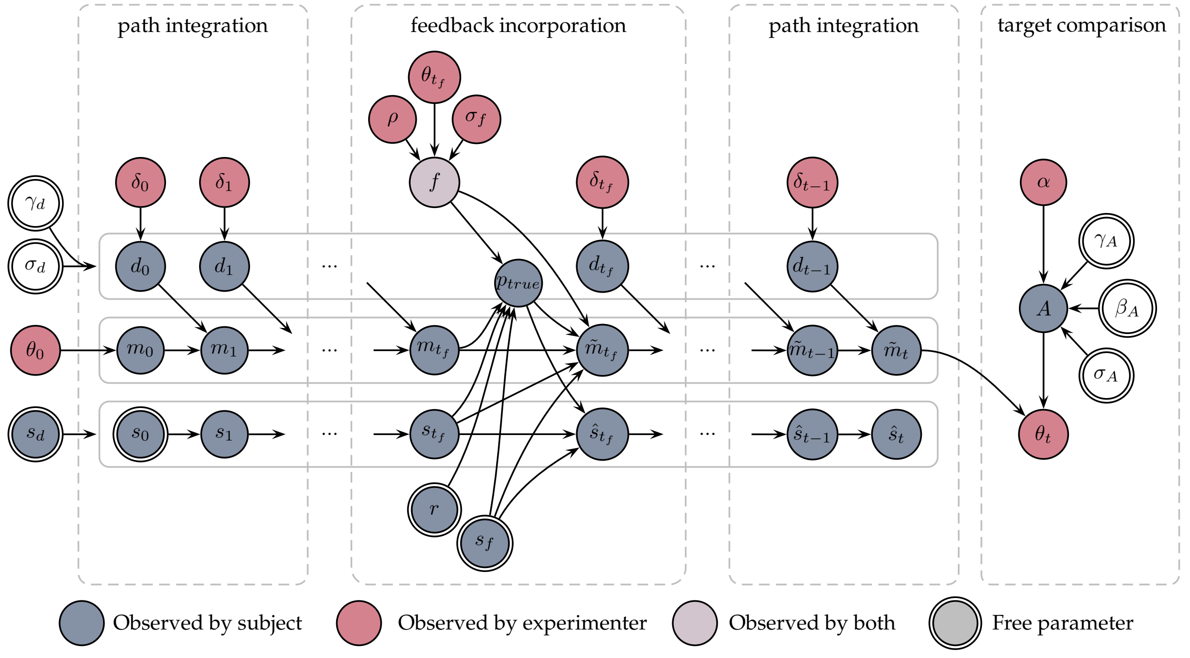

B) Mean and variance of error for the Cue Combination model

$$\mathbb{E}\left[\theta_t - \alpha\right] = \frac{1}{\gamma_d}\left((\gamma_A - \gamma_d)\alpha - K_{t_f} p_{true}(f - \gamma_d \theta_{t_f}) + \beta_A\right)$$
$$\mathbb{V}\left[\theta_t - \alpha\right] = \frac{1}{\gamma_d^2}\left(\sigma_A^2 + \left(\alpha - \left(2 - K_{t_f} p_{true}\right) K_{t_f} p_{true} \theta_{t_f}\right) \sigma_d^2\right)$$

C) Mean and variance of error for the Hybrid model

$$\mathbb{E}\left[\theta_t - \alpha\right] = \begin{cases} \frac{1}{\gamma_d}\left((\gamma_A - \gamma_d)\alpha - K_{t_f}(f - \gamma_d \theta_{t_f}) + b\right) & \text{with probability } p_{true} \\ \frac{1}{\gamma_d}\left((\gamma_A - \gamma_d)\alpha + \beta_A\right) & \text{with probability } 1 - p_{true} \end{cases}$$
$$\mathbb{V}\left[\theta_t - \alpha\right] = \begin{cases} \frac{1}{\gamma_d^2}\left(\sigma_A^2 + \left(\theta_t - \left(2 - K_{t_f}\right) K_{t_f} \theta_{t_f}\right) \sigma_d^2\right) & \text{with probability } p_{true} \\ \frac{1}{\gamma_d^2}\left(\sigma_d^2 \alpha + \sigma_A^2\right) & \text{with probability } 1 - p_{true} \end{cases}$$

Fig S3: The Cue Combination and Hybrid models. (A) Graphical representations of the parameters in the Cue Combination and Hybrid models. In the feedback incorporation stage, both models compute the probability that the feedback is true, p_{true}. They then use this probability to modulate the effect of feedback on their estimate of heading — the Cue Combination model by averaging over p_{true}, the Hybrid model with sampling from p_{true}. (B) The mean and variance of the measured error for the Cue Combination model are linear in the target angle, α, but non-linear in the prediction error, $f - \gamma_d \theta_{t_f}$ because p_{true} is non-linear in the prediction error. (C) The response distribution for the Hybrid model is a mixture of two Gaussians.

Finally, we define the participant's estimate of the prior probability that the feedback is true as a free parameter $p(\text{true}) = 1 - p(\text{false}) = r$.

Putting it all together gives the following expression for p_{true}

$$\begin{aligned} p_{true} = p(\text{true}|f, d_{1:t_f}) &= \frac{\mathcal{N}(f|m_{t_f}, s_f^2 + s_{t_f}^2)r}{\mathcal{N}(f|m_{t_f}, s_f^2 + s_{t_f}^2)r + \mathcal{U}(f)(1-r)} \\ &= \frac{1}{1 + \sqrt{\frac{s_f^2 + s_{t_f}^2}{2\pi}} \exp\left(\frac{\left(f - m_{t_f}\right)^2}{2(s_f^2 + s_{t_f}^2)}\right)\left(\frac{1-r}{r}\right)} \end{aligned} \quad \text{(S26)}$$

Note, that p_{true} depends on the square of the prediction error $(f - m_{t_f})^2$ such that when the prediction error has a large magnitude, p_{true} is small. Unfortunately this dependence requires an approximation before we can use it for model fitting. The reason is that m_{t_f} is not observed by the experimenter, only θ_{t_f}. In addition, because the dependence of p_{true} on m_{t_f} is non-linear it is not easy to average over this exactly. Instead we approximate m_{t_f} in the expression for p_{true} with its average, $\gamma_d \theta_{t_f}$, thus our approximate expression for p_{true} becomes

$$p_{true} \approx \frac{1}{1 + \sqrt{\frac{s_f^2 + s_0^2 + \theta_{t_f} s_d^2}{2\pi}} \exp\left(\frac{\left(f - \gamma_d \theta_{t_f}\right)^2}{2(s_f^2 + s_0^2 + \theta_{t_f} s_d^2)}\right)\left(\frac{1-r}{r}\right)} \quad \text{(S27)}$$

Note that s_0, s_d, and s_f do *not* appear as a ratio in the expression for p_{true}. This implies that, unlike the Kalman Filter model, all three of these parameters can be estimated from the data.

Cue Combination over p_{true} The Cue Combination model incorporates feedback by computing the mixture distribution

$$p(\theta_{t_f}|f, d_{1:t_f}) = p(\theta_{t_f}|\text{false}, d_{1:t_f}) p_{false} + p(\theta_{t_f}|\text{true}, f, d_{1:t_f}) p_{true} \quad \text{(S28)}$$

This sum is the weighted sum of the distributions from the Path Integration model, $p(\theta_{t_f}|\text{false}, d_{1:t_f})$, and the Kalman Filter model, $p(\theta_{t_f}|\text{true}, f, d_{1:t_f})$. Thus, at time t, the mean of the combined heading angle distribution is simply the weighted sum of the Path Integration estimates and the Kalman Filter estimates; i.e.,

$$\begin{aligned} \tilde{m}_t^{av} &= m_t p_{false} + \hat{m}_t p_{true} \\ &= (\gamma_d \theta_t + \nu) p_{false} + \left(\gamma_d \theta_t + K_{t_f}(f - \gamma_d \theta_{t_f}) + \epsilon\right) p_{true} \\ &= \gamma_d \theta_t + K_{t_f} p_{true}(f - \gamma_d \theta_{t_f}) + \left(1 - K_{t_f} p_{true}\right) \sum_{i=1}^{t_f - 1} \nu_i + \sum_{i=t_f}^{t-1} \nu_i \end{aligned} \quad \text{(S29)}$$

Computing the variance of $p(\theta_{t_f}|f, d_{1:t_f})$ is a little more involved. However, because this variance is unrelated to the measured behavior, we ignore it.

Target comparison As with the other models, we assume that participants stop turning when their estimate of the mean heading angle matches their noisy memory of the target, i.e. when

$$\tilde{m}_t^{av} = \gamma_A \alpha + \beta_A + n_A \tag{S30}$$

Rearranging for the measured response angle, θ_t, gives

$$\theta_t = \frac{1}{\gamma_d}\left(\gamma_A \alpha - K_{t_f} p_{true}(f - \theta_{t_f}) + \beta_A + n_A - \left(1 - K_{t_f} p_{true}\right)\sum_{i=1}^{t_f - 1} \nu_i - \sum_{i=t_f}^{t-1} \nu_i\right) \tag{S31}$$

Which implies that the error follows a Gaussian distribution with mean and variance given by

$$\begin{aligned} \mathbb{E}\left[\theta_t - \alpha\right] &= \frac{1}{\gamma_d}\left((\gamma_A - \gamma_d)\alpha - K_{t_f} p_{true}(f - \gamma_d \theta_{t_f}) + \beta_A\right) \\ \mathbb{V}\left[\theta_t - \alpha\right] &= \frac{1}{\gamma_d^2}\left(\sigma_A^2 + \left(\alpha - \left(2 - K_{t_f} p_{true}\right) K_{t_f} p_{true} \theta_{t_f}\right)\sigma_d^2\right) \end{aligned} \tag{S32}$$

The Cue Combination model has nine free parameters Table 2, all of which can be estimated from the data.

1.4 Hybrid model

Instead of averaging over the possibility that the feedback is true or false, the Hybrid model makes a decision to either incorporate the feedback (in the same way as the Kalman Filter model) or ignore it (in the same way as the Path Integration model) (Fig S3). We assume that the model makes this decision according to p_{true}, by sampling from the distribution over the veracity of the feedback. Thus with probability p_{true}, this model behaves exactly like the Kalman Filter model, with

$$\tilde{m}_t^{samp} = \hat{m}_t \tag{S33}$$

and with probability $p_{false} = 1 - p_{true}$ this model behaves exactly like the Path Integration model with

$$\tilde{m}_t^{samp} = m_t \tag{S34}$$

This implies that the distribution of errors is a mixture of two Gaussians, such that with probability p_{true}, the mean and variance of the response error are

$$\begin{aligned} \mathbb{E}(\theta_t - \alpha) &= \frac{1}{\gamma_d}\left((\gamma_A - \gamma_d)\alpha - K_{t_f}(f - \gamma_d \theta_{t_f}) + b\right) \\ \mathbb{V}(\theta_t - \alpha) &= \frac{1}{\gamma_d^2}\left(\sigma_A^2 + \left(\alpha - \left(2 - K_{t_f}\right) K_{t_f} \theta_{t_f}\right)\sigma_d^2\right) \end{aligned} \tag{S35}$$

and with probability $p_{false} = 1 - p_{true}$, the mean and variance of the response error are

$$\begin{aligned} \mathbb{E}(\theta_t - \alpha) &= \frac{1}{\gamma_d}\left((\gamma_A - \gamma_d)\alpha + \beta_A\right) \\ \mathbb{V}(\theta_t - \alpha) &= \frac{1}{\gamma_d^2}\left(\sigma_d^2\alpha + \sigma_A^2\right) \end{aligned} \tag{S36}$$

Like the Cue Combination model, the Hybrid model has nine free parameters (Table 2) all of which can be estimated from the data.

2 Bayesian decoding of target position

In the main text we assumed the following form for the biased and noisy memory of the target

$$A = \gamma_A\alpha + \beta_A + n_A \tag{S37}$$

where γ_A and β_A are the gain and bias on the memory, which lead to systematic over- and under-estimation of the target angle, and n_A is zero mean Gaussian noise with variance σ_A^2.

While this expression could simply reflect imperfect encoding of the target, here we show how this expression can be related to Bayesian decoding of a noisy, but otherwise unbiased target angle

$$A = \alpha + n \tag{S38}$$

In particular, we assume that participants are aware that their memory is imperfect and can, to some degree correct for this noise by incorporating prior knowledge about possible α angles. That is, participants use Bayesian inference compute a posterior over α given A as

$$p(\alpha|A) \propto p(A|\alpha)p(\alpha) \tag{S39}$$

Assuming both the prior and likelihood are Gaussian such that

$$\begin{aligned} p(\alpha) &= \mathcal{N}\left(\alpha|m_\alpha, s_\alpha^2\right) \\ p(A|\alpha) &= \mathcal{N}\left(A|\alpha, s_A^2\right) \end{aligned} \tag{S40}$$

where m_α is the participant's estimate of the mean of the prior distribution, s_α^2 is their estimate of the variance of the prior, and s_A^2 is the their approximation to the variance of the memory noise (i.e. their estimate of the variance of n_A).

Substituting these expressions for the likelihood and prior into Eq. S39 implies that the posterior over target angle is also a Gaussian with a mean and variance given by

$$p(\alpha|A) = \mathcal{N}\left(\alpha \;\middle|\; \frac{s_\alpha^2 A + s_A^2 m_\alpha}{s_A^2 + s_\alpha^2} \;,\; \frac{s_A^2 s_\alpha^2}{s_A^2 + s_\alpha^2}\right) \tag{S41}$$

Note that the mean of this distribution is

$$\begin{aligned} \text{mean target estimate} &= \frac{s_\alpha^2 A + s_A^2 m_\alpha}{s_A^2 + s_\alpha^2} \\ \text{variance of target estimate} &= \frac{s_A^2 s_\alpha^2}{s_A^2 + s_\alpha^2} \end{aligned} \tag{S42}$$

Note that the expression for the mean can be further related to the target by substituting $A = \alpha + n$ giving

$$\text{mean target estimate} = \left(\frac{s_\alpha^2}{s_A^2 + s_\alpha^2}\right)\alpha + \left(\frac{s_A^2 m_\alpha}{s_A^2 + s_\alpha^2}\right) + \left(\frac{s_\alpha^2}{s_A^2 + s_\alpha^2}\right) n \tag{S43}$$

Comparing this expression for the mean with Eq. S37 we can make the identifications

$$\gamma_A = \frac{s_\alpha^2}{s_A^2 + s_\alpha^2} \qquad \text{and} \qquad \beta_A = \frac{s_A^2 m_\alpha}{s_A^2 + s_\alpha^2} \tag{S44}$$

3 Fitting simulated data

We tested the validity of our model fitting procedure by fitting simulated data. This allowed us to determine whether data generated by a given model would be best fit by that model (model recovery) and whether the parameters used to generate the data could be recovered by the fitting process (parameter recovery).

3.1 Simulated data

Simulated data for each model were generated by simulating the models using the generative processes described in the main text. To ensure that the parameter values used to simulate data were in a reasonable range, we used the parameter values fit to the participants' behavior to generate parameters for the simulations. In particular, for each simulated participant we sampled each parameter randomly from the values fit to the participants. Thus, the first simulated participant could have γ_d from participant number 5, σ_d from participant number 27, and so on. In this way we ensured that the simulation parameters were in a reasonable range, but removed any correlations between the parameters in the simulation. This latter point was important for testing whether the fitting procedure induced correlations between the parameters. In all we simulated behavior from 30 participants per model (total 120 simulated participants) on the same (but scrambled) set of trials seen by real participants in the experiment. Thus the simulated data we obtained had the same number of trials as the real data set, but four times the number of participants (30 per model).

3.2 Fitting simulated data

We fit the simulated data using the same procedure used to fit the real data in the main paper. This allowed us to compute the best fitting parameter values and a BIC score for each

3.3 Model recovery

We tested the ability of the model to identify the generating model by fitting all 120 simulated data sets with all four models. Using the BIC scores for each participant, we then computed the 'confusion matrix' [3] as the fraction of times that data generated by model X was best fit by model Y, $p(\text{fit} = Y|\text{sim} = X)$ (Fig S4A). In a perfect world this matrix would be the identity matrix indicating that data generated by model X is always best fit by model X. In practice, limitations in the experiment design and fitting procedure often cause these matrices to be non-diagonal as is the case here. Nevertheless, for every model, more than 50% of the data sets generated by model X are best fit by model X.

To further help interpret the model recovery data, we also computed the 'inversion matrix' [3]. Unlike the confusion matrix, which approximates $p(\text{fit}|\text{sim})$, the inversion matrix approximates $p(\text{sim}|\text{fit})$. This we compute from the confusion matrix using Bayes rule

$$p(\text{sim}|\text{fit}) = \frac{p(\text{fit}|\text{sim})p(\text{sim})}{\sum_{\text{sim}} p(\text{fit}|\text{sim})p(\text{sim})} \tag{S45}$$

under the assumption that the prior on generating models $p(\text{sim})$ is uniform.

The inversion matrix more closely matches the inference process we face when interpreting the model fitting data in the paper. That is, we observe which model best fits each subject and must infer the model that generated it. Again we see that model recovery is good, but not perfect, such that (for example) 74% of the time that a model is best fit with the Hybrid model it was actually generated by the Cue Hybrid model.

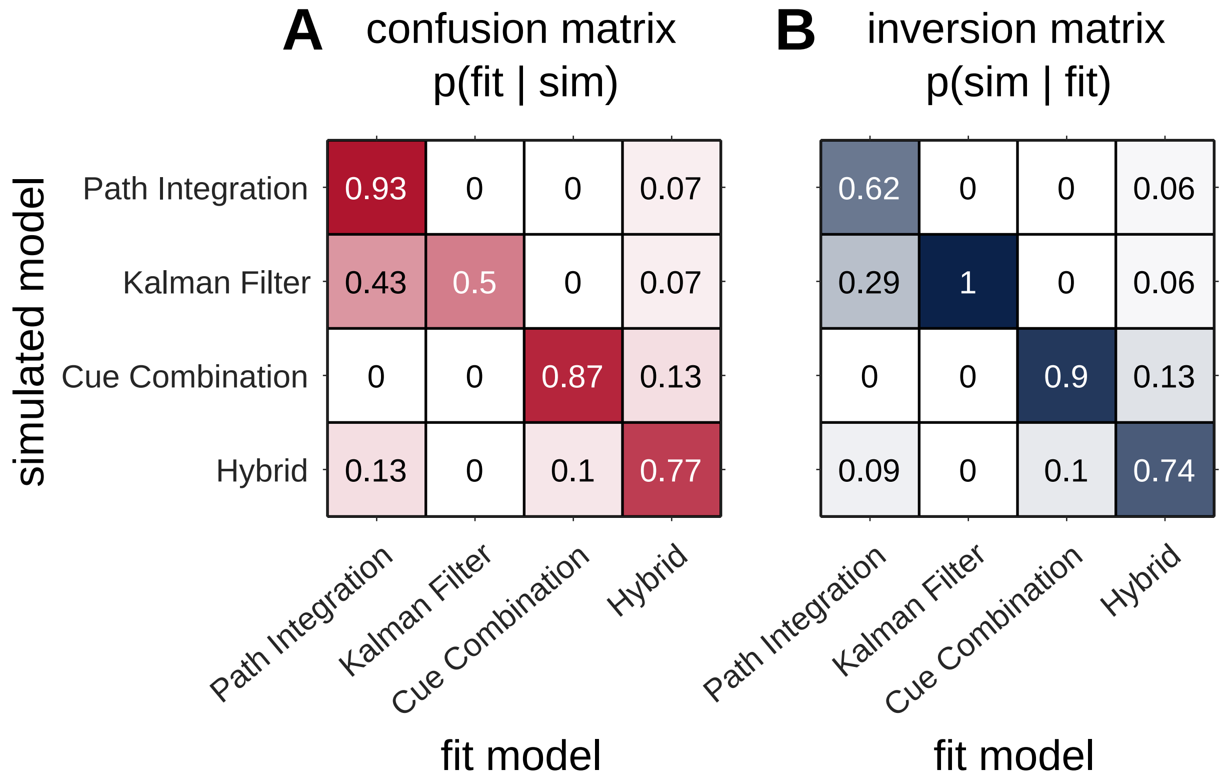

Fig S4: Confusion matrix showing the frequency with which data simulated according to each model is best fit by another model, $p(\text{fit model}|\text{simulated model})$

3.4 Parameter recovery

For the parameter recovery analysis we simulated and fit the data with the same model. First, we fit data from the Path Integration model on just the No Feedback trials S5. Parameter recovery is excellent int his case with no correlation between simulated and fit data falling below 0.84.

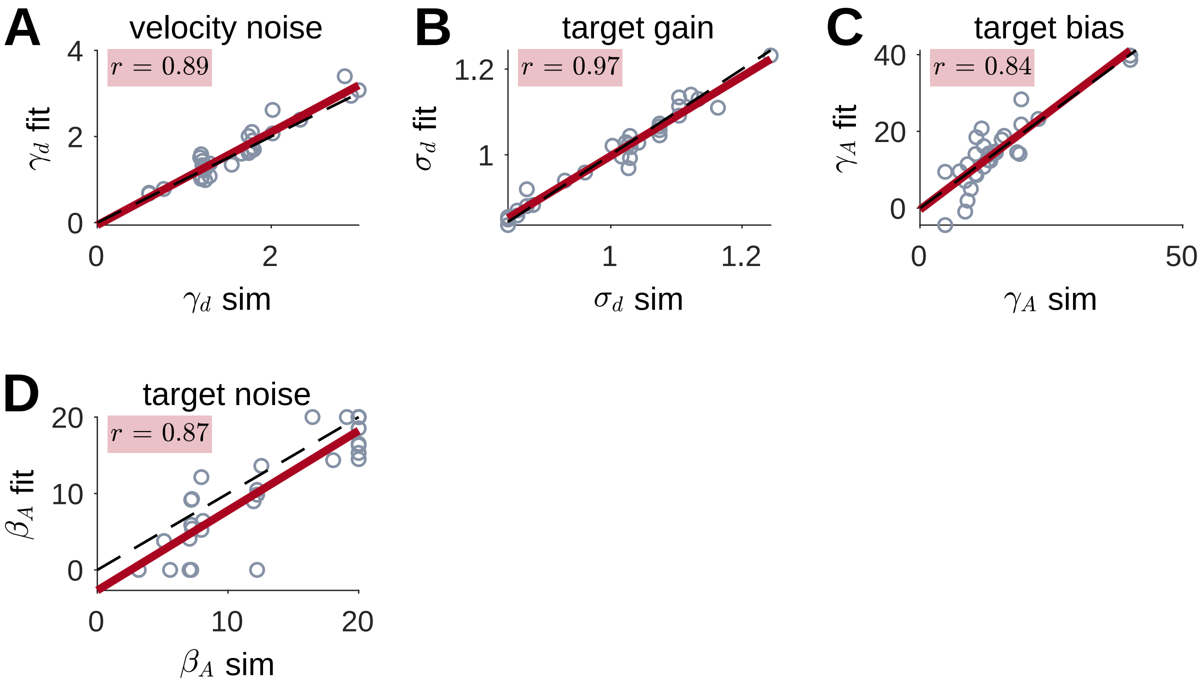

Fig S5: Parameter recovery for Path Integration model

Next we performed the same analysis for the Hybrid model, this time fitting all trials, including both the No Feedback and Feedback conditions (Fig S6). Again, parameter recovery is good for this model.

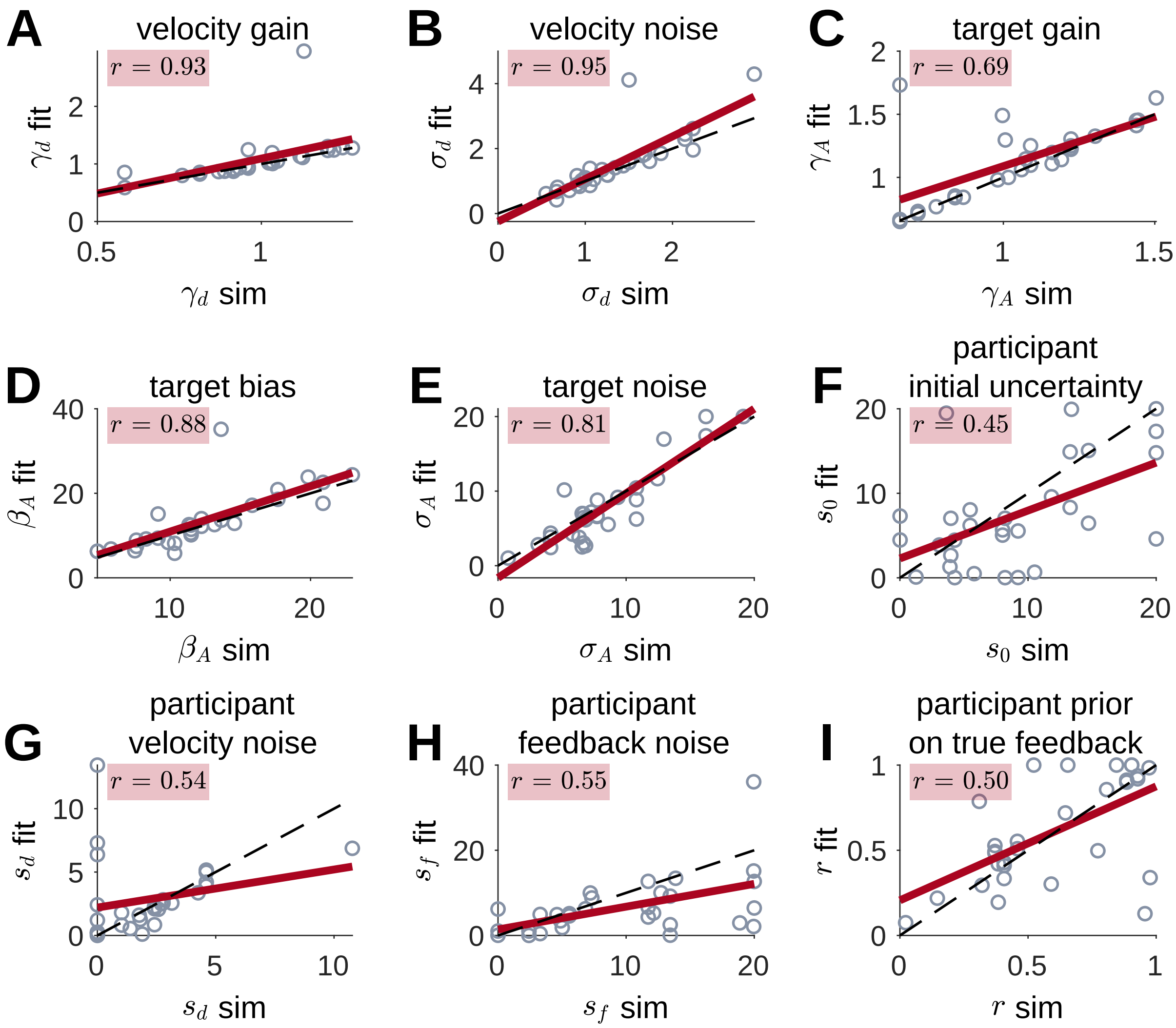

Fig S6: Parameter recovery for Hybrid model

Critically, the model fitting process did not introduce new correlations into the data set. In Fig S7 we show this for the correlations between γ_A and γ_d and σ_d.

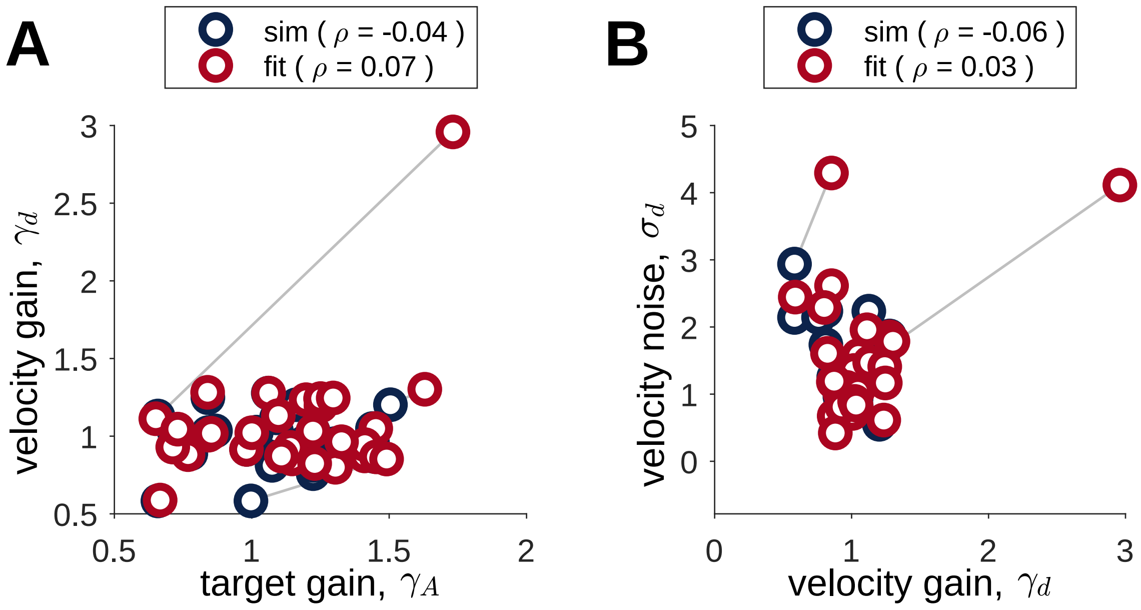

Fig S7: The fitting procedure does not induce correlations between parameters either for γ_d and γ_A (A) or γ_d and σ_d (B). When the simulation parameters (red) are uncorrelated, so are the fit parameters (blue). Gray lines connect simulated and fit parameters.

Finally, for completeness we performed parameter recovery for the remaining three models (No Feedback, Landmark Navigation, and Cue Combination model) using all trials (i.e. from the No Feedback as well as the Feedback condition). Parameter recovery was pretty good for all models Figures S8, S9, and S10.

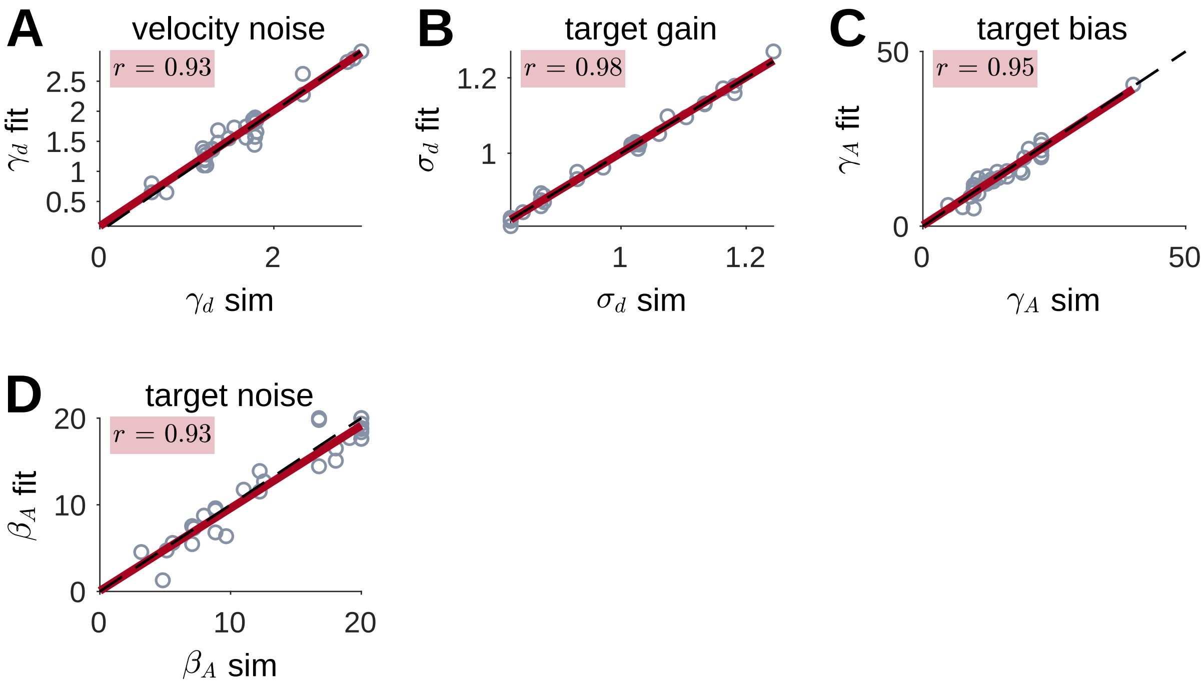

Fig S8: Parameter recovery for Path Integration model

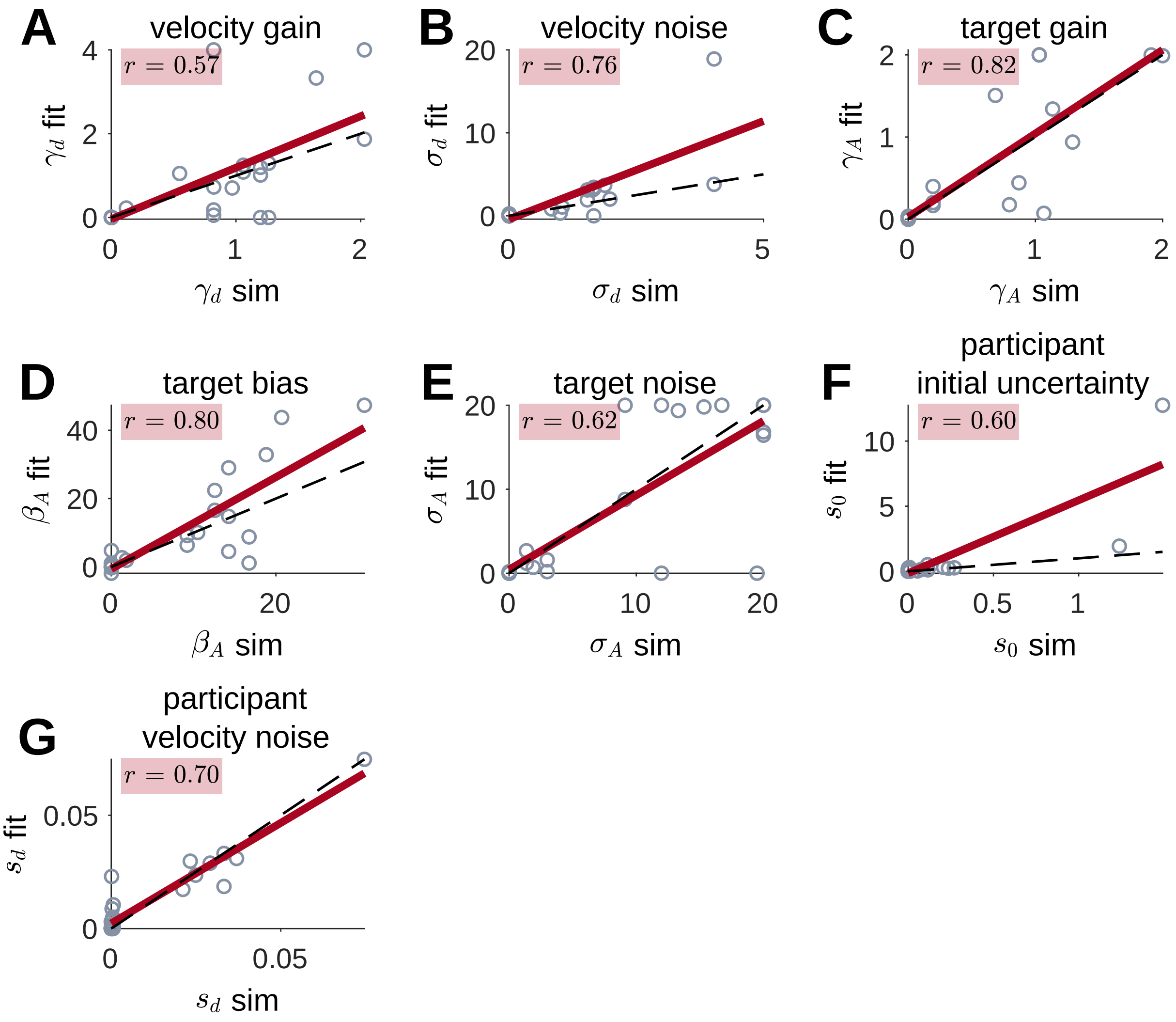

Fig S9: Parameter recovery for Kalman Filter model

4 Parameter values for the Hybrid model

Fit parameter values for the Hybrid model are shown in Fig S11. Like the model-free measures of behavior, there was considerable variability in the parameter values across participants. Thus, while the group average of the velocity gain was close to 1 (mean $\gamma_d = 1.01$) individuals varied from systematically under-weighting velocity ($\gamma_d < 1$) to systematically over-weighting it ($\gamma_d > 1$). All participants exhibited noise in their velocity coding process, with $\sigma_d = 1.38$ on average. This latter result suggests that the variance of the uncertainty in location from path integration grows rapidly, at around 1.38 times the rotation angle. At this rate of growth, the noise in path integration will swamp the signal in less than one turn.

Similarly suboptimalities were observed in the coding of the target. Like the velocity gain, the group average of the target gain was close to 1 (mean $\gamma_A = 1.04$), there was considerable variation between people from systematic under-weighting ($\gamma_A < 1$) to systematic over-weighting of the target ($\gamma_A > 1$). In addition, as in the No Feedback condition, all participants were biased towards over-estimating the target (mean $\beta_A = 13.0$ degrees) and had considerable noise in the target coding process (mean $\sigma_A = 7.5$ degrees).

There were also considerable individual differences in participants' inference parameters: s_0, s_d, s_f, and r. Most participants underestimated the feedback noise (mean $s_f = 8.68$ versus the true value in the experiment of $\sigma_f = 30^o$). Conversely, the group average of the prior probability was more accurate (mean $r = 0.69$ which is remarkably close to the true value of $\rho = 0.7$, although again there was considerable variability across the group.

The individual differences in s_0, s_d, and s_f lead to considerable variability in the Kalman gain across participants and (in some participants) across trials (Fig 12). Some participants show almost no variation across trials (left and right sides of Fig 12), while others show large variability across trials (middle participants in Fig 12). This pattern can be explained by recalling the equation for Kalman gain

$$K_{t_f} = \frac{s_0^2 + s_d^2 \theta_{t_f}}{s_f^2 + s_0^2 + s_d^2 \theta_{t_f}} \tag{S46}$$

This equation implies that participants with both small and large noise in their path integration process (i.e. $s_d \approx 0$ or $s_d \gg s_f$ and s_0) will have approximately constant Kalman gain across trials. When the velocity noise is small ($s_d \approx 0$),

$$K_{t_f} \approx s_0^2 / (s_0^2 + s_f^2) \tag{S47}$$

which is a constant between 0 and 1 depending on the ratio of participants initial uncertainty s_0 to their estimate of feedback noise s_f. This is the case for participants on the left hand side of Fig 12.

When the velocity noise is large ($s_d \gg s_f$ and s_0)

$$K_{t_f} \approx s_d^2 \theta_{t_f} / s_d^2 \theta_{t_f} = 1 \tag{S48}$$

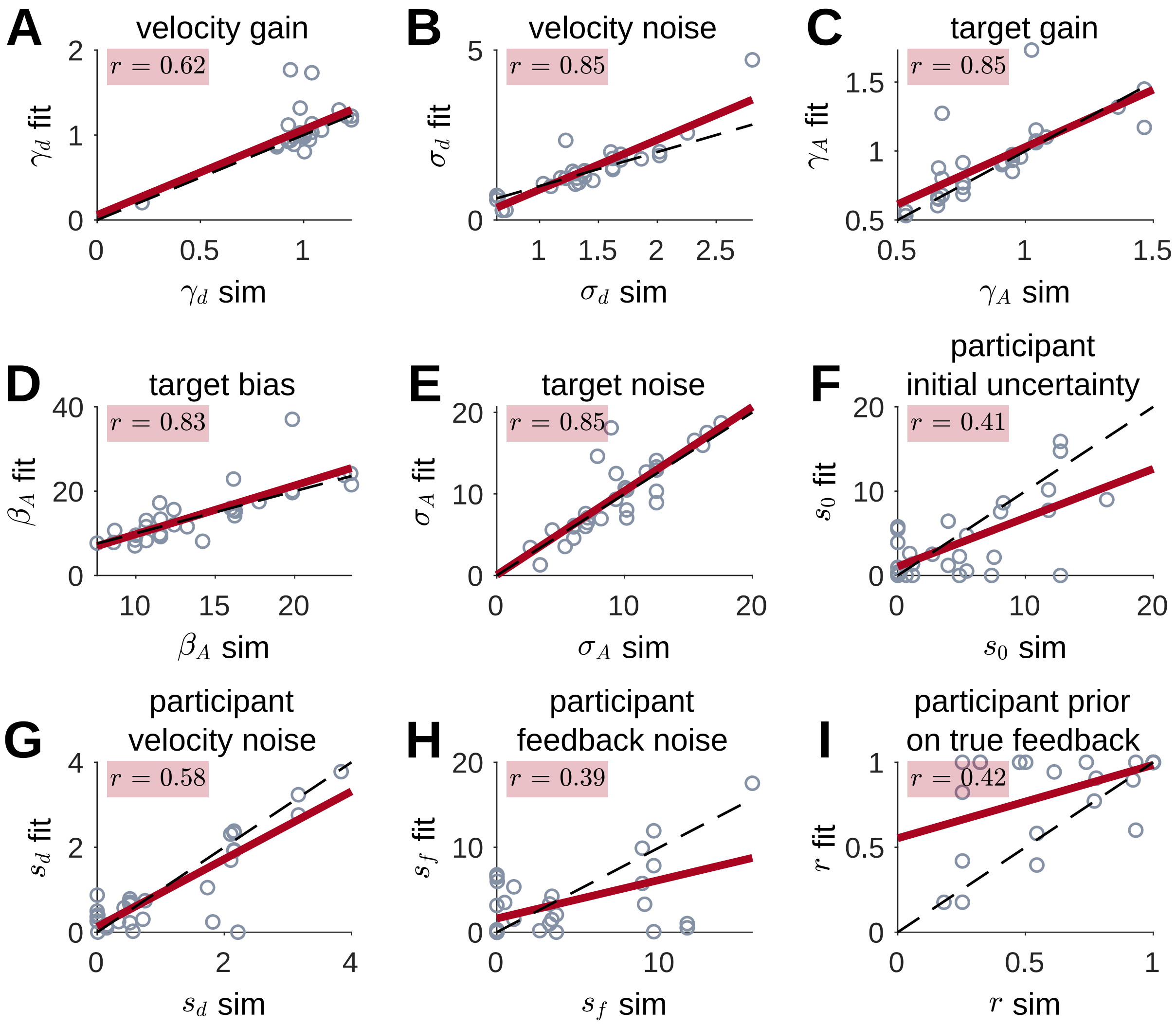

Fig S10: Parameter recovery for Cue Combination model

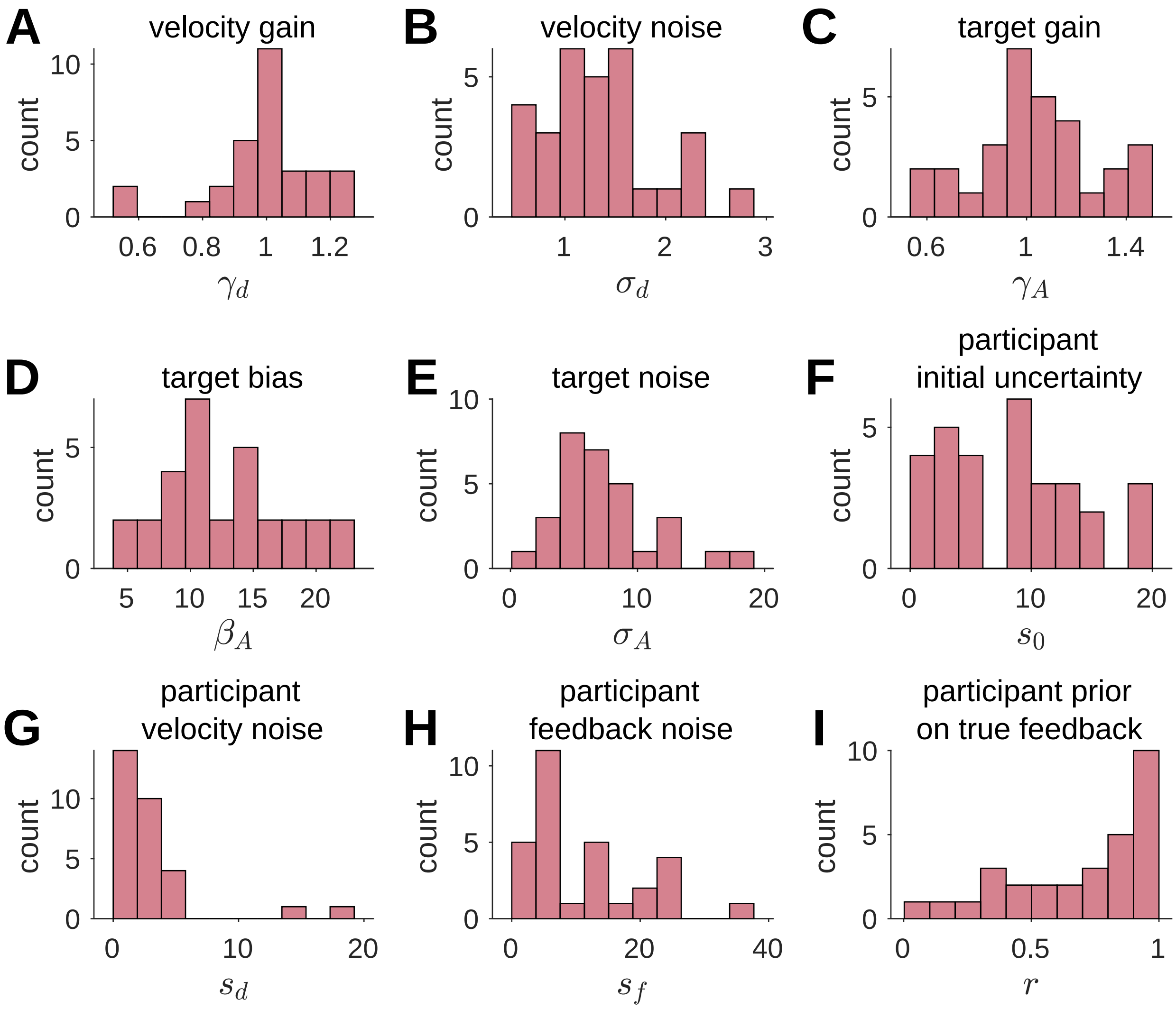

Fig S11: Best fit parameters for the Hybrid model

That is the Kalman gain is a constant with value equal to 1. This is the case for participants on the right hand side of Fig 12.

For participants whose velocity noise is intermediate in value, the uncertainty in their estimate of heading at the time of feedback is close to their estimate of the feedback noise giving them a Kalman gain between 0 and 1 that varies considerably depending on the exact value of the true heading angle at feedback θ_{t_f}.

4.1 Correlations between parameters

Finally we consider the correlations between fit parameter values (Fig S12). Although our relatively small sample size limits the power of this analysis, we find three significant correlations and two near-significant correlations after Bonferroni correction for multiple comparisons.

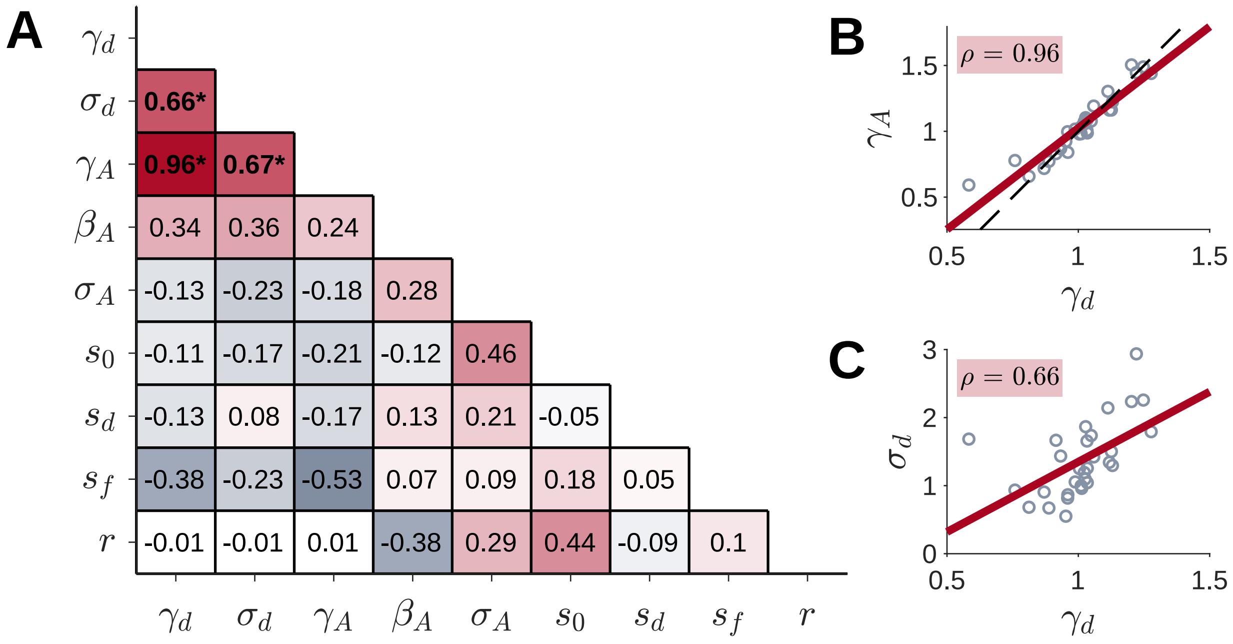

Fig S12: Correlations between parameters in the Hybrid model. (A) Spearman correlation coefficients for all nine parameters. * indicates $p < 0.05$ after Bonferroni correction for multiple comparisons. (B) The correlation between the gain on velocity, γ_d, and the gain on the target γ_A is near perfect. The red line corresponds to the linear least squares fit, the black dashed line to the equation $\gamma_A = 2\gamma_d - 1$. (C) γ_d also correlates with the velocity noise, σ_d, as does γ_A (not plotted).

The most striking of these correlations is the near-perfect correlation ($r = 0.96$) between the target gain γ_A and velocity gain γ_d (Fig S12B). As shown by the parameter recovery

analysis in Fig S7, this correlation is not an artefact of the fitting procedure. Instead we believe that this reflects a redundancy in the model whereby the same gain process that contributes to people's imperfect coding of the target also contributes to imperfect coding of velocity. Intriguingly, the correlation in Fig S12B is almost perfectly described by the equation

$$\gamma_A = 2\gamma_d - 1 \tag{S49}$$

which is the dashed black line in Fig S12B.

This relationship can cause displacement biases of target location in the direction of motion, which is a phenomenon in perception called *representational momentum* [4–6] . This displacement, characterized as a memory bias, is directly influenced by velocity and has a linear relationship for small changes in velocity [7, 8]. Thus, we speculate that this linear relationship between this deviation from perfect gain (i.e. gain = 1) in memory and velocity is the derivative equivalent to representational momentum. However, exactly why the slope of this relationship should be 2 is a mystery to us at this stage.

The other significant (and near significant) correlations also involve γ_d and γ_A with the velocity noise σ_d and the participant's estimate of feedback noise s_f. Because γ_d and γ_A are so tightly coupled, it is not surprising that their correlations with σ_d and s_f are almost identical and we focus only on the correlations with γ_d in Fig S12.

For velocity noise, a positive correlation with γ_d (Fig S12C) can be understood if the noise in the velocity estimate occurs *before* the gain is applied. This is consistent with modifying the equation for the noisy velocity to be

$$d_i = \gamma_d(\delta_i + \nu_i) \tag{S50}$$

where the standard deviation of the noise is k, which relates to the standard deviation of the noise in the original model as $\sigma_d = \gamma_d \times k$.

Finally, while asserting the null comes with serious caveats with such a small sample size, we note one correlation that was not significant. In particular, we note that s_d and σ_d are only weakly correlated. Perfect Bayesian inference would have these equal, as participants use their estimate of their own velocity noise to optimally integrate feedback. If our model is correct, then this suggests that participants may not have a good estimate of their own path integration noise.

5 Model fit for all subjects

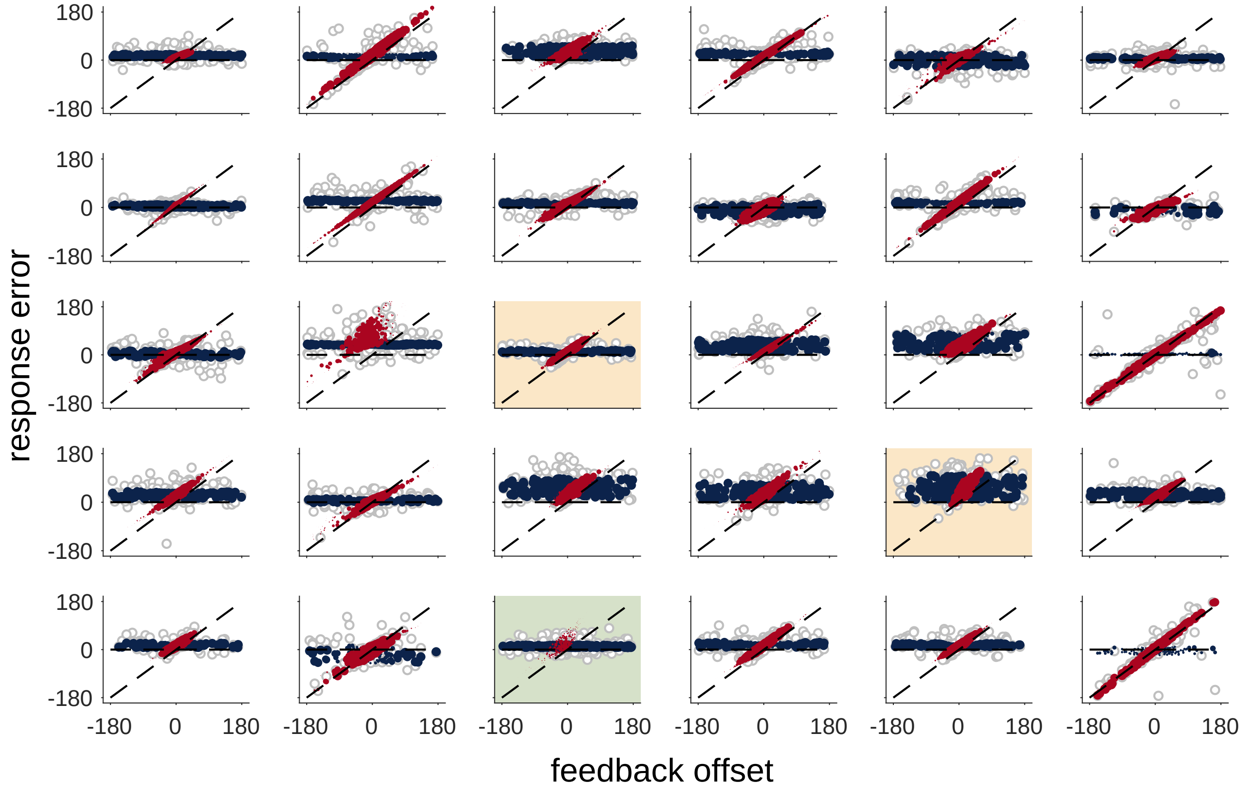

Fig S13: Comparison between data and model for all participants. The background color represents which model was the best fit(Fig 13) for that participant: Hybrid model (white), path integration (green) and Cue Combination (yellow).

6 Confidence Rating Correlations

At the end of each trial, participants rated their confidence by adjusting the angle ς in Fig 1D and E. We found no correlation between any participant's confidence rating and their angle error (Fig S14), which is consistent with previous work showing that people are relatively bad at judging their own errors [9, 10]. Interestingly some participant's confidence rating does correlate with target angle Fig S15. In other words, these participants feel less and less confidant as they continue rotating. One possible explanation that these participants not only estimating target location but rather they are calculating full posterior distribution [11]. Indeed some of these participants show a significant correlation with the posterior variance calculated from the Hybrid model Fig S16. However, with the larger individual differences, it is hard to make an exact conclusion.

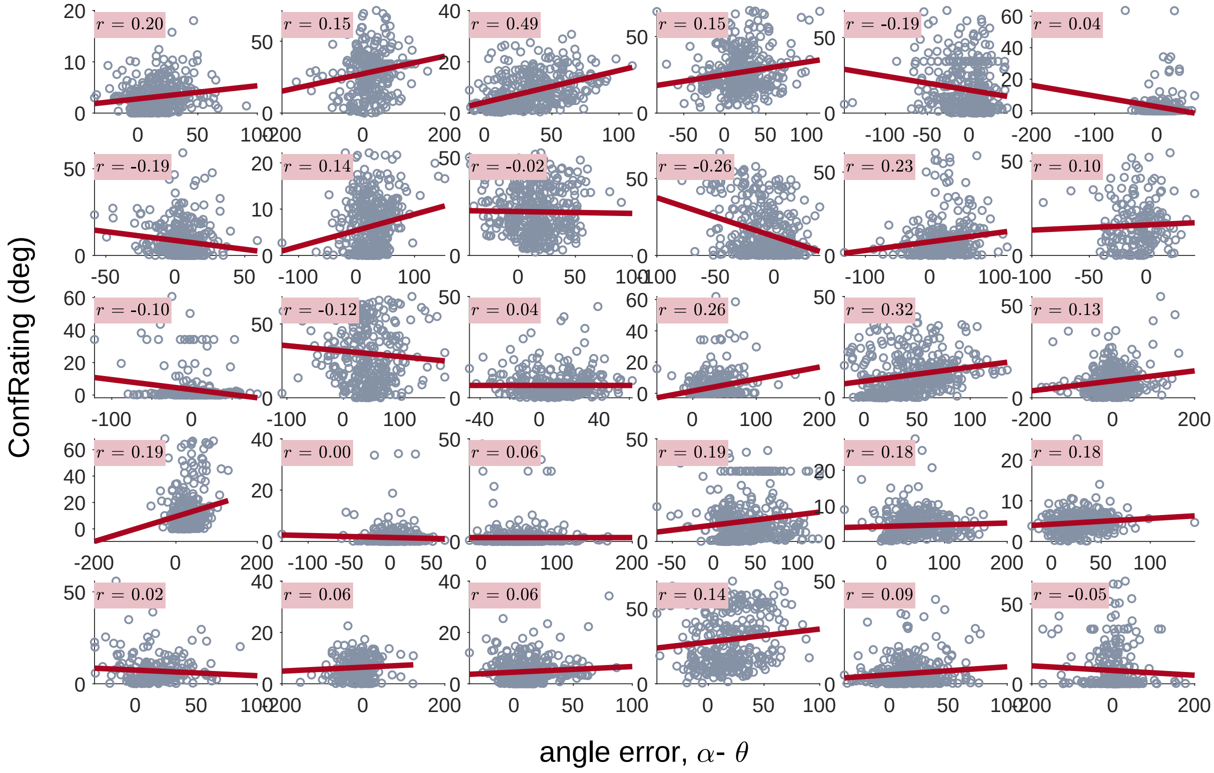

Fig S14: Subject's confidence rating plotted against their angle error

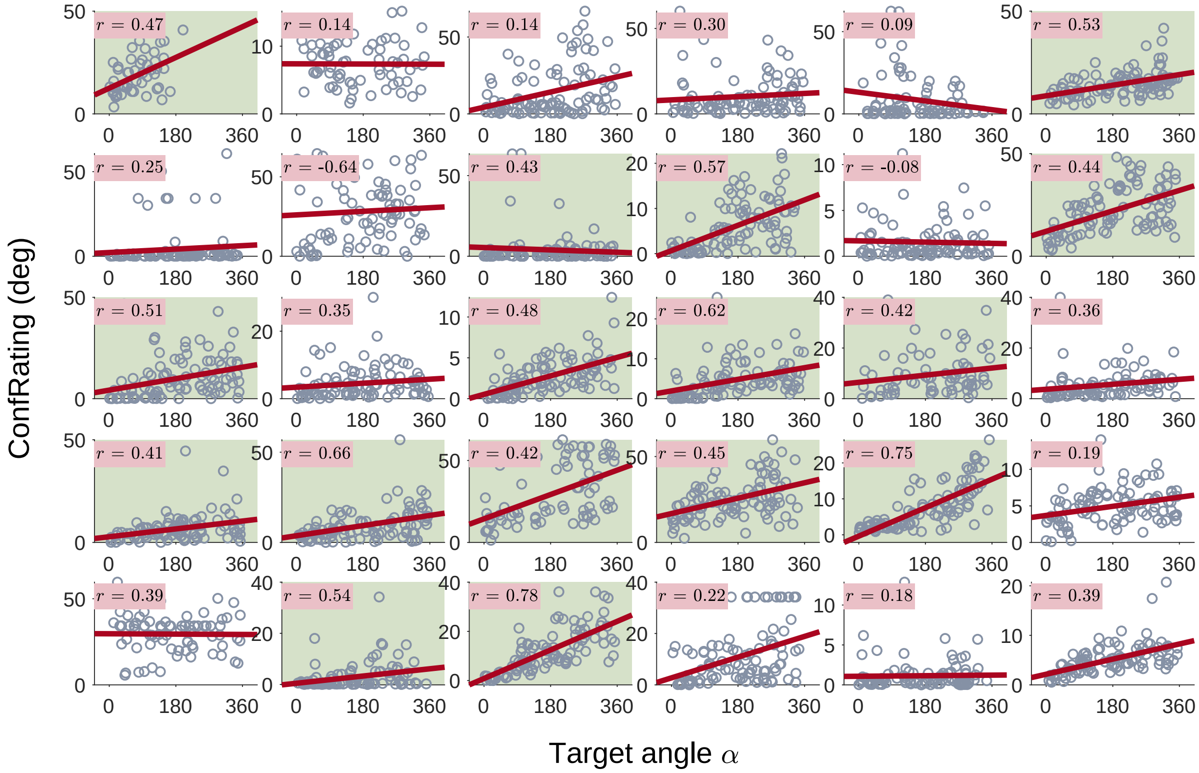

Fig S15: Subject's confidence rating plotted against target location. Green: $r >\geq 0.4$

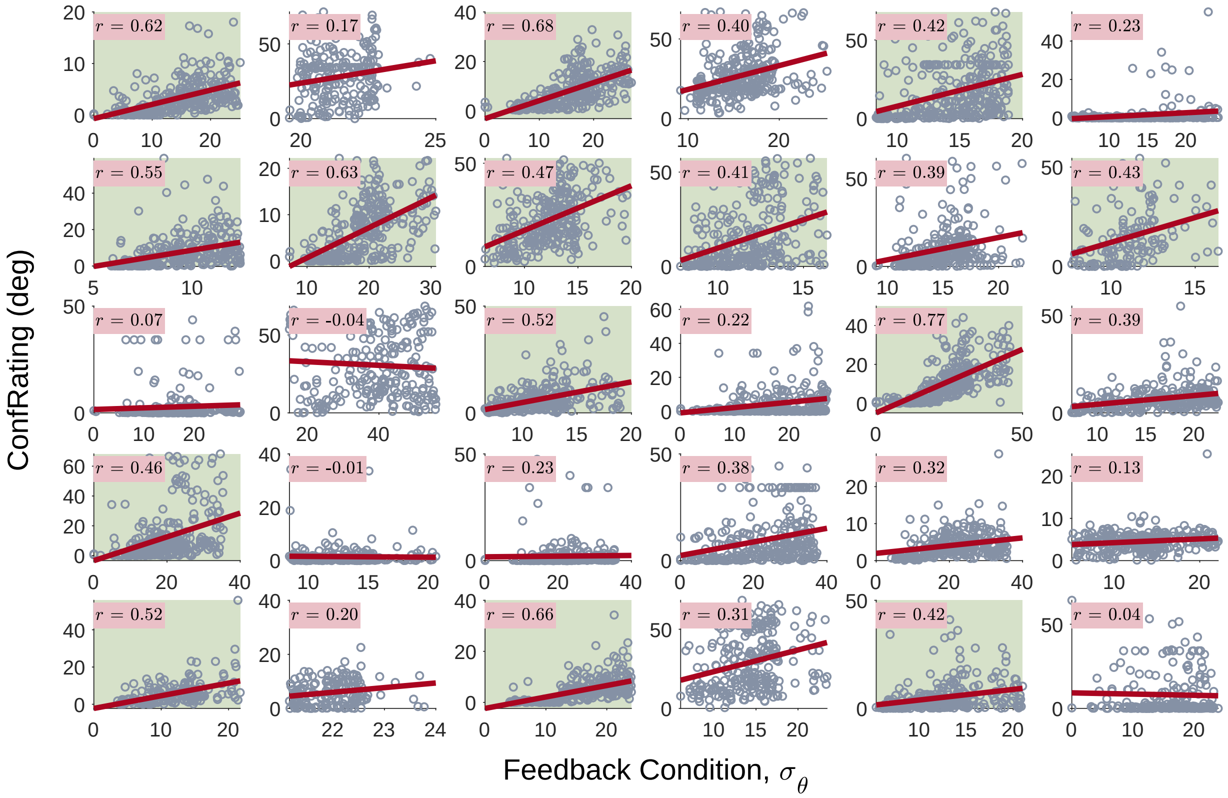

Fig S16: Subject's confidence rating plotted against the posterior variance Eq. 13 and S36

References

[1] Kalman RE. A new approach to linear filtering and prediction problems. J Basic Eng. 1960;82(1):35–45.

[2] Harootonian SK, Wilson RC, Hejtmánek L, Ziskin EM, Ekstrom AD. Path integration in large-scale space and with novel geometries: Comparing vector addition and encoding-error models. PLoS computational biology. 2020;16(5):e1007489.

[3] Wilson RC, Collins AG. Ten simple rules for the computational modeling of behavioral data. eLife. 2019;8:e49547.

[4] Freyd JJ, Finke RA. Representational momentum. Journal of Experimental Psychology: Learning, Memory, and Cognition. 1984;10(1):126.

[5] Freyd JJ, Finke RA. A velocity effect for representational momentum. Bulletin of the Psychonomic Society. 1985;23(6):443–446.

[6] Hubbard TL. Representational momentum and related displacements in spatial memory: A review of the findings. Psychonomic Bulletin & Review. 2005;12(5):822–851.

[7] Munger MP, Minchew JH. Parallels between remembering and predicting an object's location. Visual Cognition. 2002;9(1-2):177–194.

[8] Munger MP, Dellinger MC, Lloyd TG, Johnson-Reid K, Tonelli NJ, Wolf K, et al. Representational momentum in scenes: Learning spatial layout. Memory & cognition. 2006;34(7):1557–1568.

[9] Rahnev D, Denison RN. Suboptimality in perceptual decision making. Behavioral and Brain Sciences. 2018;41.

[10] Kruger J, Dunning D. Unskilled and unaware of it: how difficulties in recognizing one's own incompetence lead to inflated self-assessments. Journal of personality and social psychology. 1999;77(6):1121.

[11] Sanders JI, Hangya B, Kepecs A. Signatures of a statistical computation in the human sense of confidence. Neuron. 2016;90(3):499–506.
