# Supplementary material for: Combination and competition between path integration and landmark navigation in the estimation of heading direction
Source: PLoS Comput Biol. 2022 Feb 10;18(2):e1009222. doi: 10.1371/journal.pcbi.1009222 (PMC8865642; doi:10.1371/journal.pcbi.1009222)
Supplement: Attachment — Submitted filename: Ekstrom_Competition_PCOMPBIOL-D-21-01111_review.pdf [file pcbi.1009222.s018.pdf]

Review of PCOMPBIOL-D-21-01111, Harootonian, Ekstrom, & Wilson
"Combination and competition between path integration and landmark navigation in the estimation of heading direction"

This is a very strong manuscript on an important problem with conflicting results in the existing literature: how does the navigation system combine idiothetic information from path integration with allothetic information from the visual scene? The authors have complete command of the literature on path integration and cue combination in navigation and clearly motivate the current experiment. The writing is admirably clear and the logic is well-developed. The authors collect a prodigious amount of data and use it to test four nested models of cue combination and competition, with strong model-fitting and careful analysis of individual differences. I do have one major reservation that may involve further data collection, and a number of other comments below that the authors should address in a revision.

1. The Kalman gain, which determines the relative weight of visual feedback to path integration, is large (>.75) for most subjects, and close to 1.0 for half the subjects (Fig. 12). This is the case even though the authors only provide minimal visual feedback: the *duration* of the feedback was very brief (300 ms). In addition, the symmetry of the virtual environment may have created some visual ambiguity (bookcases on every wall, parallel tables flanking the subject, etc.). It seems likely that with longer feedback durations the Kalman gain will approach 1, the navigation system would be purely competitive, and visual information would dominate. In that case the visual feedback would reset the path integrator, as observed in rodents (Etienne, et al., 2004) and humans (Zhao & Warren, *PS*, 2015), until the visual offset gets very large.

   Thus, I'm concerned that the present findings do not reflect typical navigation conditions, and I'm not persuaded that they would generalize to such conditions. In effect, the authors are asking the navigator to solve an unusual problem, which may invoke complex decision-making processes (the hybrid model with 9 free parameters) that are not normally involved in navigation. I would urge the authors to collect additional data with longer feedback durations to see if their results generalize to more typical viewing conditions.

2. In comparing their findings to previous results (Lines 436-438), the authors say, "Thus, in the same task, participants appeared to switch from cue combination to cue competition as the offset grew larger, exactly what we observe in our experiment, and what is predicted by the Hybrid model." The trouble is that previous cue competition results found complete dominance by visual landmarks, whereas the authors report complete dominance by path integration, at larger offsets. Why do they observe the opposite? Perhaps because they have minimized the visual feedback (see comment #1).

3. In this connection, Zhao & Warren (*Cognition*, 2015) manipulated the stability of environmental landmarks, and found that this dramatically influenced what the authors describe as the Kalman gain: when visual landmarks did not shift position for many trials, subjects relied completely on the landmarks and rejected path

integration (gain = 1); when landmarks changed position markedly for many trials, subjects rejected the landmarks and relied on path integration (gain = 0). This is further evidence of cue competition, and the dominant cue flips depending on the environmental stability.

4. By reducing the homing problem to one dimension (orientation or head direction), the authors may have oversimplified the problem. Mou and colleagues (Mou & Zhang, Cogntion, 2014; Zhang & Mou, JEP:LMC, 2016; Zhang, Mou & Du, JEP:LMC, in press) have argued that information from path integration and visual landmarks is combined differently for self-localization (position and head direction) than for homing (returning to the start position). Thus, it's not clear whether the present results for cue combination in head direction will generalize to the navigation task of homing. The authors should address this question of generalization.
5. How do the cue combination and hybrid models described by the authors differ from Bayesian robust cue integration (Knill, 2007, *Journal of Vision, 7*, 1-24; Shams & Beierholm, 2010, *TiCS, 14*, 425-432)? Is this what the authors have in mind in Lines 455-462? At some point they should compare their models to this previous account.
6. The authors report the number of free parameters for the Path Integration model (5) on Line 332. They should likewise report the number of free parameters for each of the other models in the main text, in the results section for the Feedback Condition (Line 351 ff). I assume that the BIC computation penalized each model for its free parameters, correct?

<u>Details</u>

- I find the symbolic notation to be strikingly unintuitive. Subscripts don't correspond to idiothetic and visual signals, *f* represents both "feedback angle" and "false", *d* represents velocity while *v* represents noise, and I'm not sure what subscript *t* represents (time, turn, or the temporal derivative? Not target, because for some reason that's represented by *A*). At best, the symbols should be rethought; at least, add a table of symbols.
- Line 47: References should be [23, 25-27]
- Fig. 1b,c: Why is the virtual room so symmetric, with bookcases on every wall, two parallel tables, stone walls on every side, etc?
- Line 97: Say how participants were "guided" through a rotation, with a haptic signal.
- Lines 106-108: This haptic vibration cues the direction of rotation – it's not actually "feedback" about anything (until it *ends*). So perhaps call it a "haptic signal".
- Line 160: "The virtual environment stayed in the same orientation" is ambiguous. The same allocentric or egocentric orientation?

- Line 175: The path integration process is said to integrate “vestibular” cues, but I think this should be “idiothetic” because the participant is actively turning.
- Line 275-276: I can’t make this into a grammatical sentence.
- Line 304: Delete “be”
- Fig. 11 caption: Explain what the open, black, and red circles and the small black dots represent.
