# Supplementary material for: Combination and competition between path integration and landmark navigation in the estimation of heading direction
Source: PLoS Comput Biol. 2022 Feb 10;18(2):e1009222. doi: 10.1371/journal.pcbi.1009222 (PMC8865642; doi:10.1371/journal.pcbi.1009222)
Supplement: Attachment — Submitted filename: RR_Final.pdf [file pcbi.1009222.s019.pdf]

PLOS-CompBio

Reviewer Response for "Combination and competition between path integration and landmark navigation in the estimation of heading direction"

Review 1

R1.1.

The Kalman gain, which determines the relative weight of visual feedback to path integration, is large (>.75) for most subjects, and close to 1.0 for half the subjects (Fig. 12). This is the case even though the authors only provide minimal visual feedback: the duration of the feedback was very brief (300 ms). In addition, the symmetry of the virtual environment may have created some visual ambiguity (bookcases on every wall, parallel tables flanking the subject, etc.). It seems likely that with longer feedback durations the Kalman gain will approach 1, the navigation system would be purely competitive, and visual information would dominate. In that case the visual feedback would reset the path integrator, as observed in rodents (Etienne, et al., 2004) and humans (Zhao & Warren, PS, 2015), until the visual offset gets very large.

Thus, I'm concerned that the present findings do not reflect typical navigation conditions, and I'm not persuaded that they would generalize to such conditions. In effect, the authors are asking the navigator to solve an unusual problem, which may invoke complex decision-making processes (the hybrid model with 9 free parameters) that are not normally involved in navigation. I would urge the authors to collect additional data with longer feedback durations to see if their results generalize to more typical viewing conditions.

We thank the reviewer for this critical analysis of our task design, results, and modeling. We would like to address each point the reviewer made.

We agree with the reviewer that our virtual room may be too symmetrical, and have changed "... distinct landmarks…" to "proximal landmarks" on line 377. Our intention in creating a symmetrical environment was to avoid biasing left and rightward turns. While there may have been some visual ambiguity, we believe this effect would be minor as there were no verbal reports of confusion about the content of the feedback. In addition, because feedback was a "glimpse" of the room it was a dynamic viewing of the room and many participants saw, on average, over 20° of the room during the 300ms.

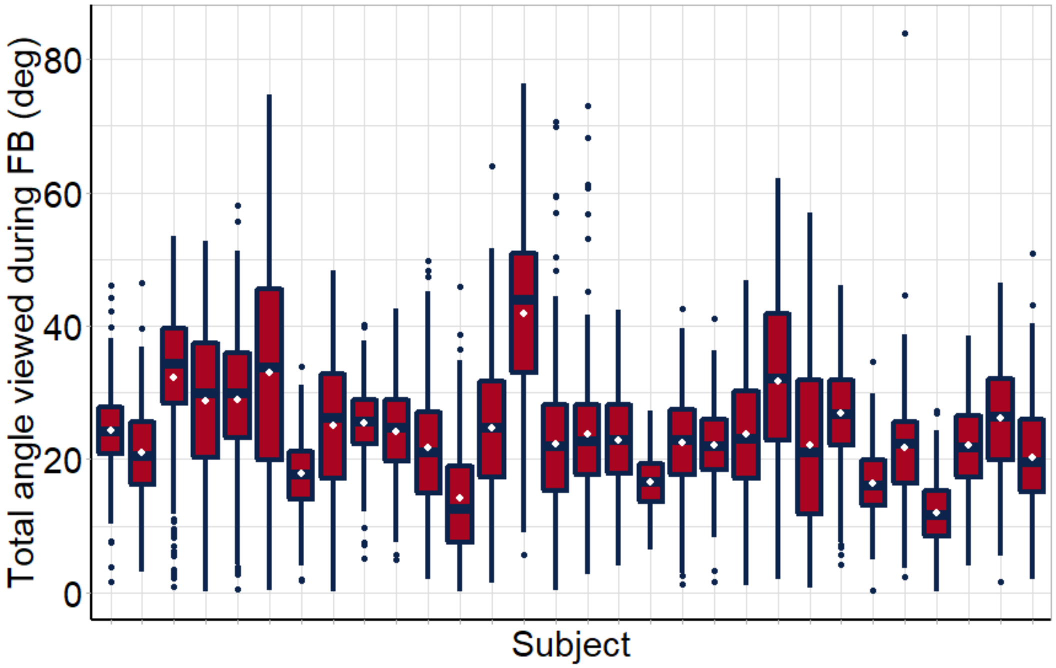

Furthermore, we found no correlation between the Kalman gain and the feedback viewing amount across all trials and all participants.

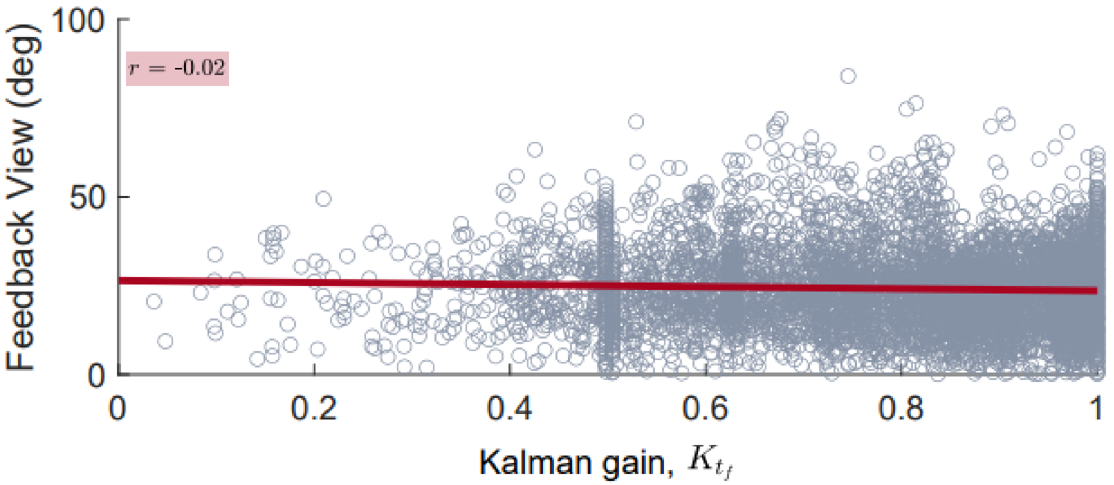

We agree with the reviewer that typical navigation conditions may be different as the duration of visual information hinders the assessment of its reliability. However, there are situations where navigational decisions have to be made with limited time and information. For example, while driving you may be unsure of whether the building just you saw was your destination or not and must make a quick decision to turn around or not. While this example is in a different situation than our task, we believe that the underlying individual differences in our task propagate into more general decisions made in the example. We attempted to model and capture *sources* of individual differences with our model, which resulted in a model with 9 free parameters. We hope that our model and modeling framework can be used to improve future navigation studies.

Finally, we agree with the reviewer's intuition that if visual feedback was increased from 300ms, then the Kalman gain for all participants would most likely increase. We do plan to run the suggested experiment in addition to expanding our task design to include rotations and translations. However, because the first author has moved to another university, and because of ongoing difficulties running in-person studies due to COVID, we would ask the reviewer and editor to reconsider the need for additional data in this paper, which we believe makes a valuable contribution already by presenting the models and new task. We hope the significant change in the Discussion which highlights the reviewer's concern would be a satisfactory alternative.

"One explanation for this difference between our results and Zhao and Warren's could be the amount of time that feedback was presented for. In \cite{zhao2015you}, the offset feedback was presented continuously, whereas in our task thefeedback was presented for only 300ms. Thus, participants in Zhao and Warren's experiment may have been more confident that the visual feedback was correct which, by equation \ref{eq:kalmanGain}, would lead to a Kalman gain close to 1. Consistent with this idea, in a different study, Zhao and Warren \cite{zhao2015environmental} found, using a catch trials design, that visual landmark reliability increased with environmental stability. In addition, they observed individual differences in cue switching behavior with most individuals showing no cue switching behavior at all. This suggests that for continuous stable visual feedback the Kalman gain will approach 1 for most

participants. Interestingly our visual feedback was not continuous and was only moderately stable, yet several participants had a Kalman gain close to 1 (Eq. \ref{eq:kalmanGain}). Given these results, an increased visual feedback duration would likely result in more reliance on visual cues and hence a general increase in the Kalman gain. A critical question for future work will be to ask how the Kalman gain changes as a function of viewing duration and a range of different environmental stabilities."

R1.2.

In comparing their findings to previous results (Lines 436-438), the authors say, "Thus, in the same task, participants appeared to switch from cue combination to cue competition as the offset grew larger, exactly what we observe in our experiment, and what is predicted by the Hybrid model." The trouble is that previous cue competition results found complete dominance by visual landmarks, whereas the authors report complete dominance by path integration, at larger offsets. Why do they observe the opposite? Perhaps because they have minimized the visual feedback (see comment #1).

We apologize for any confusion over this point. The particular study the sentence in question was referring was Zhao and Warren 2015 PS. For ease of reference we paste the key figure for our purposes here (Supplementary Fig 1 from their paper).

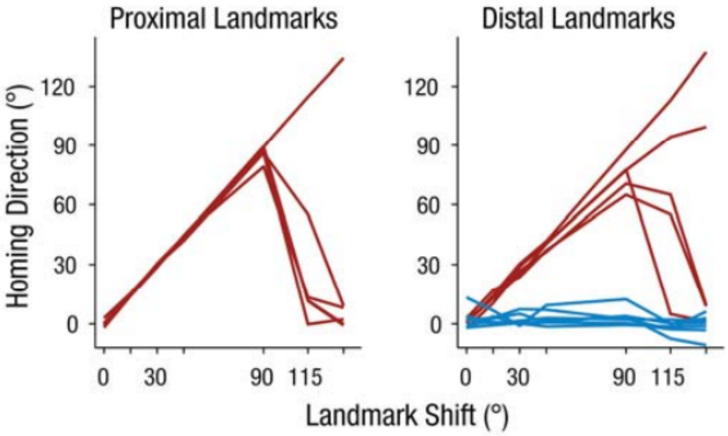

In this figure, the "landmark shift" plays the role of our "feedback offset" and "homing direction" is similar to our "response error." When landmark shifts are large the homing direction returns to 0, consistent with participants ignoring visual feedback for large shifts. As we understand this figure, this behavior *is* consistent with the Hybrid model with parameters set such that the Kalman gain is 1.

We do agree with the reviewer that these Zhao and Warren results suggest that when visual feedback is used it completely dominates path integration (i.e. Hybrid model with Kalman gain = 1). To acknowledge this point, we have added the following to the Discussion:

"Our findings in support of a Hybrid model may help to explain the mixed reports in the literature regarding cue combination. Specifically, some studies report evidence of cue combination \cite{nardini2008development,chen2017cue,foo2005humans,zhang2020cue} while others find evidence for others cue competition \cite{zhao2015you,chrastil2019vision}. One set of studies using a similar experimental paradigm involves the short-range homing task of Nardini and colleagues and shows evidence for both cue combination and competition depending on the conditions tested \cite{nardini2008development}. In this task, participants picked up three successive objects in a triangular path and returned them after a delay. During the return phase of the task, the experimenters manipulated the visual feedback to induce a 15

degree mismatch with the body-based cues. When both visual and body-based cues were present, Nardini et al. found that the variance of the response was smaller than when navigation relied on only one cue, consistent with the combination of visual and body-based cues in a Bayesian manner. However, when Zhao and Warren \cite{zhao2015you} increased the offset from 15 to 135 degrees, they found that participants based their estimate of location either entirely on the visual cues (when the offset was small) or entirely on the body-based cues (when the offset was large), taking this as evidence for a cue-competition strategy. Thus, in the same task, participants appeared to switch from visual cue integration to path integration as the offset grew larger. Such behavior is consistent with Hybrid model, albeit with a Kalman gain that is equal to 1, which is slightly different to what we observed in our experiment (Fig. \ref{fig:kalmanGain}).”

R1.3.

In this connection, Zhao & Warren (*Cognition*, 2015) manipulated the stability of environmental landmarks, and found that this dramatically influenced what the authors describe as the Kalman gain: when visual landmarks did not shift position for many trials, subjects relied completely on the landmarks and rejected path integration (gain = 1); when landmarks changed position markedly for many trials, subjects rejected the landmarks and relied on path integration (gain = 0). This is further evidence of cue competition, and the dominant cue flips depending on the environmental stability.

We thank the reviewer for pointing this study out. We agree with the reviewer on the relevance and importance of Zhao & Warren (*Cognition*, 2015) and have included the discussion about environmental stability in tandem with our discussion about visual cue duration.

"One explanation for this difference between our results and Zhao and Warren's could be the amount of time that feedback was presented for. In \cite{zhao2015you}, the offset feedback was presented continuously, whereas in our task we feedback was presented for only 300ms. Thus, participants in Zhao and Warren's experiment may have been more confident that the visual feedback was correct which, by equation \ref{eq:kalmanGain}, would lead to a Kalman gain close to 1. Consistent with this idea, in a different study, Zhao and Warren \cite{zhao2015environmental} found, using a catch trials design, that visual landmark reliability increased with environmental stability. In addition, they observed individual differences in cue switching behavior with most individuals showing no cue switching behavior at all. This suggests that for continuous stable visual feedback the Kalman gain will approach 1 for most participants. Interestingly our visual feedback was not continuous and was only moderately stable, yet several participants had a Kalman gain close to 1 (Eq. \ref{eq:kalmanGain}). Given these results, an increased visual feedback duration would likely result in more reliance on visual cues and hence a general increase in the Kalman gain. A critical question for future work will be to ask how the Kalman gain changes as a function of viewing duration and a range of different environmental stabilities."

R1.4

By reducing the homing problem to one dimension (orientation or head direction), the authors may have oversimplified the problem. Mou and colleagues (Mou & Zhang, Cogntion, 2014; Zhang & Mou, JEP:LMC, 2016; Zhang, Mou & Du, JEP:LMC, in press) have argued that information from path integration and visual landmarks is combined differently for self-localization (position and head direction) than for homing (returning to the start position). Thus, it's not clear whether the present results for cue combination in head direction will generalize to the navigation task of homing. The authors should address this question of generalization.

We agree that this is a limitation, and apologize if it was not clearly acknowledged in initial submission. We have added the following to the Discussion to address this point.

"One limitation of this work is that we have focused only on rotation, ignoring translation completely. While this approach has the advantage of simplifying both the analysis and the task (e.g. removing the risk of participants accidentally walking into a real-world wall during their virtual navigation), it may be that we are missing something crucial. Indeed, related to this point, Mou and colleagues \cite{mou2014dissociating,zhang2017piloting,zhang2020cue} have argued that estimates from path integration and visual landmarks are combined differently depending on whether the task is self-localization (position and head direction) or homing (returning to the start position). Thus, while in the rest of the Discussion, we focus on the general implications of our work, a key for future work will be to expand our experimental paradigm and model to account for translational and well as rotational movements."

R1.5

How do the cue combination and hybrid models described by the authors differ from Bayesian robust cue integration (Knill, 2007, *Journal of Vision, 7*, 1-24; Shams & Beierholm, 2010, *TiCS, 14*, 425-432)? Is this what the authors have in mind in Lines 455-462? At some point they should compare their models to this previous account.

We thank the reviewer for point us to this literature. Indeed the Robust cue integration is similar to our Hybrid Model and have included the it in line 510:

Similar to Robust cue integration \cite{knill2007robust}, when faced with a large feedback offset, instead of computing the full posterior distribution over heading, participants collapse this bimodal distribution to a unimodal distribution centered on the estimate from path integration or landmark navigation, ignoring the less reliable cue.

However, Knill 2007 found that a Bayesian mixture model is sufficient in explaining the behavior in their study. We have cited this study and along with other fully-Bayesian alternative models on line 514. Furthermore, we have expanded on an experimental design to be able to distinguish between these two interpretations.

A key question for future work will be to distinguish between these two interpretations of the task. Does sampling occur at the time of feedback causing a collapse of the posterior distribution to one mode and a loss of information? Or does sampling occur later on and without the collapse of the posterior? Both interpretations lead to identical behavior on the Rotation Task. However, a modified version of the task should be able to distinguish them. One way to do this would be with two turns and two sets of visual feedback rather than one. In this task, participants would turn first to landmark A (e.g. the door) and then to landmark B (e.g. the window). The turn to landmark A would be identical to the task in this paper, with a brief flash of offset feedback along the way. After reporting their estimate of the location of landmark A, and crucially without feedback as to whether they were correct, participants would then make a second turn to landmark B. This second turn would also include a flash of visual feedback. If this second flash aligned with one mode of the bimodal posterior it should reinforce that mode if participants kept track of it. However, if they collapsed their posterior to a single mode, a flash at the other mode would have less effect. Thus, in principle, the two accounts could be distinguished.

R1.6

The authors report the number of free parameters for the Path Integration model (5) on Line 332. They should likewise report the number of free parameters for each of the other models in the main text, in the results section for the Feedback Condition (Line 351 ff). I assume that the BIC computation penalized each model for its free parameters, correct?

We apologize to the reviewer for the confusion. Indeed the BIC values do penalize for the number of free parameters. We have added the following to the result section before discussing the BIC values. In addition, we also note that to test the performance of BIC for model comparison we performed a "model recovery" analysis (Supplementary Figure 4) in which we asked whether simulated data from each model was best fit by the model that generated the data. As shown by the relatively diagonal confusion matrix, this model comparison metric works well.

"We computed Bayes Information Criterion (BIC) scores for each of the models for each of the participants, which penalizes the likelihood values of each model fit by the number of free parameters. The Hybrid and Cue Combination models have 9 free parameters, while the Kalman filter and Path integration models have 8 and 5 free parameters respectively (Table 2)"

In addition, we have added Table 2 (which was previously in the Supplementary Material) to the main text. This table outlines which parameters are in which model.

R1.Minor

1. I find the symbolic notation to be strikingly unintuitive. Subscripts don't correspond to idiothetic and visual signals, *f* represents both "feedback angle" and "false", *d* represents velocity while *v* represents noise, and I'm not sure what subscript *t* represents (time, turn, or the temporal derivative? Not target, because for some reason that's represented by *A*). At best, the symbols should be rethought; at least, add a table of symbols.

We apologize for the difficulty here. We tried our best with the notation - unfortunately it is hard to avoid this complexity with so many variables and we do not feel that a different notation would be more intuitive. However, we have tried to make the existing notation more accessible by including (1) a table of all variables (Table 1), (2) a "Summary of Models" subsection at the end of the Models section of the Methods, and (3) a table of the free parameters for all models (Table 2, which was previously in the Supplementary Material). This is in addition to the figures always naming the parameters as well as displaying the associated symbol (e.g. Figs 1-4, 7, 8, 9, 11, 12).

2. Line 47: References should be [23, 25-27]

We thank the reviewer for catching this.

3. Fig. 1b,c: Why is the virtual room so symmetric, with bookcases on every wall, two parallel tables, stone walls on every side, etc?

We thank the reviewer for this critical question. We intended to control for left/right handed turns by having similar saliancy for the objects in the room. We have added the following sentence to the Methods to address this point:

We controlled the saliency of the decorations for left and right-handed rotations by placing identical objects in similar vertical positions (Fig. \ref{fig:task}C).

4. Line 97: Say how participants were "guided" through a rotation, with a haptic signal.

Done. We added the following:

"...participants were first guided, with a haptic signal from hand held controllers, through a rotation…"

5. Lines 106-108: This haptic vibration cues the direction of rotation – it's not actually "feedback" about anything (until it *ends*). So perhaps call it a "haptic signal".

Done. We have changed our wording to haptic signal.

6. Line 160: "The virtual environment stayed in the same orientation" is ambiguous. The same allocentric or egocentric orientation?

We apologize for the confusion. The environment stayed in the same egocentric orientation. Hence if they moved their head the visuals from the headset would not change. We modified the text to "same egocentric orientation."

7. Line 175: The path integration process is said to integrate "vestibular" cues, but I think this should be "idiothetic" because the participant is actively turning.

We agree with the reviewer and have changed vestibular to idiothetic.

8. Line 275-276: I can't make this into a grammatical sentence. + Line 304: Delete "be"

Done. Thanks for catching this typo.

9. Fig. 11 caption: Explain what the open, black, and red circles and the small black dots represent.

We have updated the caption of Figure 11 to the following:

"*Four participants' data (open grey dots) are overlaid hybrid model's mean responses when the model assumes the feedback is true (red) and false (blue). The size of the dots corresponds to the probability that the model samples from a distribution with this mean, i.e. p_{true} for red and $1-p_{true} = p_{false}$ for blue.*"

Review 2

R2.1

For Figure 10 and the BIC model comparison, is there a cutoff number that indicates strongly in favor of the Hybrid model (akin to how the Bayes Factor scores are interpreted)? I would imagine that many of these are quite low and could be considered weak evidence. Currently the authors are taking anything that favors the Hybrid model as evidence, but that could be overinterpreting the findings.

We thank the reviewers for their questions. The BIC value is a relative measure of goodness of fit and unlike the Bayes Factor there is no standard as the values can range widely depending on the model and data type. However, we did conduct a series of different model comparisons, by testing the parameter and model recovery Fig S4-6,S8-10. Parameter recovery analysis shows that simulation parameters can be recovered when fitting data with the same model that generated it. This suggests that fit parameter values bear some relation to the ground truth. Model recovery analysis shows that the best fitting model for simulated data is the model that actually simulated the data. This suggests that model recovery is fairly robust.

In addition, we fixed the x-axis label for Fig10A.

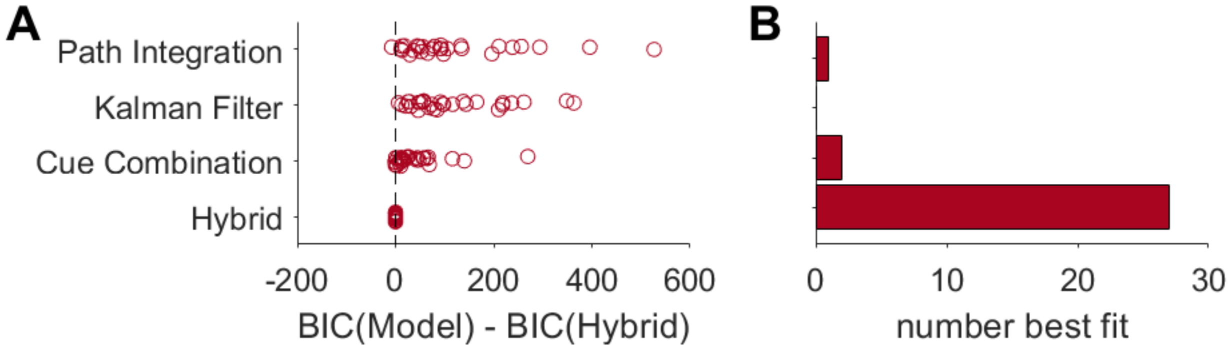

R2.2

Path integration model: This model assumes that everything that is happening is during the response phase. It sounds like it allows for error during encoding, which is the remembered angle. That's fine if you are focusing just on the response portion, but you should probably make these kinds of assumptions explicit. For example, when reading the Appendix, it says "As they turn…" at first I thought this was for the encoding turn (or for both), but I think it's just the response turn. I think the confusion arises because there are two processes: path integration and target comparison. Presumably you are integrating on the encoding turn as well, so that makes it a bit confusing here.

We apologize to the reviewer for the confusion and we agree this should be explicitly said. We added the following in the Modeling section of the main text.

"Note, the following models focus exclusively on the Retrieval portion of the task. The target location from the Encoding portion of the task is modeled in Supplementary Section 2"

In addition, we also changed *"As they turn…"* in the supplementary to *"During retrieval, we assume that participants receive vestibular cues about their angular velocity as they rotate."*

R2.3

Intro: allocentric visual information is not just landmarks. Optic flow is a major allocentric visual cue to path integration, so that distinction should be made clear.

We thank the reviewer for helping us clarify this point. We originally tried to implicitly address this by referring to idiothetic without opic flow as “body-based idiothetic cues”. However, we agree with the reviewer that an explicit note will help clarify for the general reader. We added the following in the introduction:

“This multisensory input comes in two forms: idiothetic cues, from one's own rotations and translations (including body-based cues from the vestibular, proprioceptive, and motor efferent copy systems, as well as visual optic flow), and allothetic cues, from the environment (usually visual landmarks). In this paper we investigate how information from body-based idiothetic and visual allothetic cues are integrated for navigation.”

R2.4

If the consistent feedback range is about 60 degrees, then for the inconsistent trials wouldn't about 1/6 of the time in the random sampling the feedback was actually consistent? Does this affect the interpretation of the Hybrid model at all?

We thank the reviewer for this question. Because the model is based on only the feedback angle, and not the hidden variable as to whether the feedback was consistent or not, the model (and subject) would see such feedback as being indistinguishable from consistent feedback. As such this does not affect the interpretation of the Hybrid model.

R2.5a

For models that incorporate path integration: How often is this path integration sampling occurring, or is it continuous? Mostly I'm looking at the schematic in Figure 2 (which is very helpful!), but wondering whether you are modeling this continuously even though the figure is discrete.

Sorry for any confusion here. We opted for the relative simplicity of discrete time, both in Figure 2 and the Equations (e.g. using subscript *t*, e.g. in m_t which tends to imply discrete time, versus parentheses, e.g. m(t) which tends to imply continuous time), to illustrate the path integration process. However, the model is readily extended to continuous time. For the model fitting, we only needed to consider values of mean and uncertainty at two time points (the time of feedback and at the end of the turn).

R2.5b

Also, is the path integration always (leaky) and underestimating (as would be suggested by many models and experimental evidence), or is it random in its over and underestimates? The schematic suggests that it can be under and some points and over at others. Is this really what we see experimentally? This applies to the path integration sections of the other models.

In fact, all three possibilities exist in the model. In particular, consider equation 2 from the main paper:

$$d_t = \gamma_d \delta_t + \nu_t$$

In this equation the model's estimate of velocity d_t is based on a scaled (by \gamma_d) and noisy (\nu_t) version of the true velocity \delta_t. If the gain factor \gamma_d = 1 and the noise is finite, then the estimate of position will on average be correct, but will sometimes randomly be ahead and sometimes behind the true location. If the gain factor is less than 1, then the velocity estimates will be systematically too low and the model will underestimate position.

Finally if the gain factor is greater than 1, then velocity estimate will be too high and people will overestimate location.

As shown below, we see both a mean underestimation, no bias, and overestimation in the data, with more participants overestimating the angle

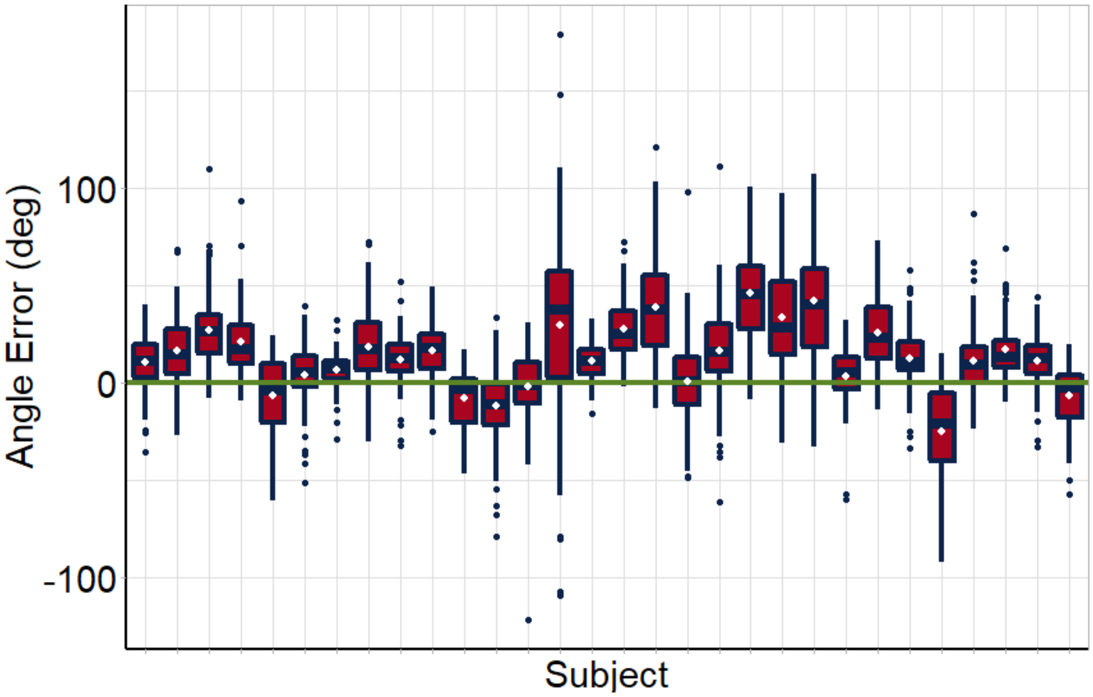

The white dots are the mean angle error for each subject for the no-feedback condition. The green horizontal line is centered at 0. To capture the systematic errors of underestimation and overestimating in path integration we used the velocity gain parameter (γ_d) to capture both.

Consistent with this finding, the fit parameter values suggest \gamma_d varies across the population around 1 and the velocity noise, captured by the variance parameter, \sigma_d, is non-zero (panels A and B of Supplementary Figure S11).

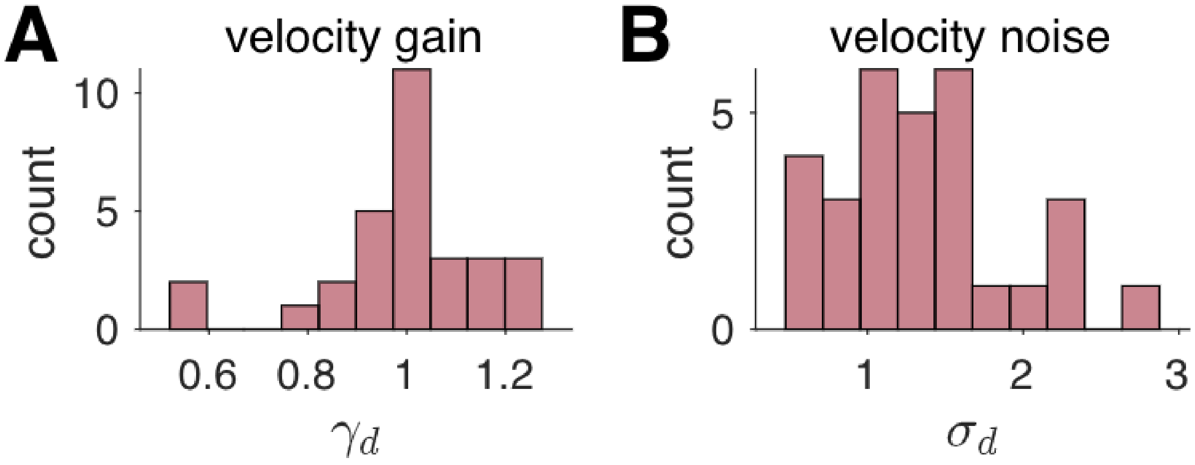

R2.6

The Discussion could talk a bit more about path integration models in general and how these results compare

We have added the following to the Discussion pointing to some of the neural models of path integration / cue combination in the head direction system.

"Previous computational models based on line attractor neural networks produce behavior that is almost identical to the Hybrid model, combining cues when they are close together and picking one when they are far apart \cite{zhang1996representation,touretzky2005attractor,wilson2009neural,jeffery2016optimal,sun2018analysis,sun2020decentralised}. Moreover, recent findings in fruit flies suggest that, at least in insects, such networks may actually be present in the brain \cite{kim2017ring}. Investigating the link between the Hybrid model and its neural implementation should be a fruitful line of research."

R2.Minor

1. Figure 1: caption says 100 trials of FB, but the figure itself indicates 300 trials. Based on the text later on, I think the caption is reversed.

We thank the reviewer for pointing out this typo, we have corrected the trial numbers.

2. I don't think it is specifically said, so the authors should make the prediction explicit in the path integration model that they expect no difference in response angle between feedback and no feedback conditions (per angle)

We thank the reviewer for this concern and added the following in the Kalman Filter model section.

"Initial path integration, is identical to the Path Integration model (used in No Feedback trials)."

3. For some clarification, the Hybrid model says that it goes with either Path Integration or the Kalman Filter on a trial-by-trial basis? So any given trial is not diagnostic, but the collection of trials will tell you that it is the Hybrid?

This is correct, without multiple trials there would be no bimodal behavior to fit.

4. For the combined estimate in Fig 4, where does this long tail come from? It doesn't look Gaussian.

That is correct, because the Cue Combination model combines the estimates (and distributions) of the Path Integration model and the Kalman Filter model, the resulting posterior is a mixture of two Gaussians and can (when the mismatch between the Path Integration and Kalman Filter estimates is large) be bimodal.

5. Figure 7, is the most negative slope person missing trials?

We thank the reviewer for this question. Yes, there are only 49/100 no feedback trials plotted for this participant. In addition after processing and filtering the head direction data from the HTC Vive, we ended up with a total of 189 trials (49noFB and 140FB). For processing we filter out trials that have imperfect tracking during a trial. This includes stutters, gaps or lags in the tracking. We believe the cause is due to a low battery in the wireless receiver, however we are not sure. For trials with longer target goals the participant spent longer time turning and increased the probability of inaccurate tracking for those trials. However, we decided to include

the participant data as the inaccurate recording of the tracking does not influence the participant's experience.

6. Figure 8 needs more caption. What do the counts mean?

We apologize to the reviewer for not being clear. The counts are the number of participants, whose fitted parameter values fall between the bin width. The larger the spread the less generalization and larger individual differences in the parameter values across participants. We added the following to Figure 8's caption.

"*Histograms show the distribution parameter values across participants. The counts are the number of participants, whose fitted parameter values fall within each bin.* "

7. Figure 11 needs more caption. What are the colors and filled and open dots?

We apologize to the reviewer for our lack of detail and have updated the caption of Figure 11 to the following:

"*Four participants' data (open grey dots) are overlaid hybrid model's mean responses when the model assumes the feedback is true (red) and false (blue). The size of the dots corresponds to the probability that the model samples from a distribution with this mean, i.e.* p_{true} *for red and* $1-p_{true} = p_{false}$ *for blue.*"

8. Line 470: "future gold" should be "future goal"

Thank you for pointing out this typo.

Supplement:

9. For Table S1, I think the headings reflect older names, should have Hybrid and Kalman Filter listed. What does the check mark mean?

Thanks for catching this typo. We have corrected it in the updated version of this table, which we also moved to the main text (as Table 2) in response to Reviewer 1.

The checkmarks represent when a parameter is included in the model, we have included the following in the table caption to address this point.

"Parameters, their ranges and values, in the different models. The presence of a parameter in a model is indicated by either a check mark (when it can be fit on its own), a ratio (when it can be fit as a ratio with another parameter), or $=1$ when it takes the value 1."

10. For the Bayesian decoding of target position, what is the basis for the assumption that people know their memory is imperfect and that they incorporate prior knowledge?

In the strictest interpretation, our model does not assume Bayesian decoding of the target location. Instead, via equation 4 we wanted to encode the possibility that the target is not included correctly with a possibility for a gain error (\gamma_A), a fixed bias (\beta_A) and random noise n_A.

$$A = \gamma_A \alpha + \beta_A + n_A$$

One interpretation of this equation, which we go into detail on in Supplementary Section 2, is that equation 4 could arise from Bayesian decoding of a noisy memory of the target. However, we did not mean to assert that this is the only way such imperfect target encoding could occur. To try to tone down the Bayesian interpretation, we have added the following in Supplementary Section 2

"While this expression could simply reflect imperfect encoding of the target, here we show how this expression can be related to Bayesian decoding of a noisy, but otherwise unbiased target angle"

11. Would the possible alpha angles be related to what angles they had experienced in the experiment?

The true target angles, \alpha, were sampled randomly and independently on each trial, there was there no dependence between trials such that previous angles did not affect future angles.

12. In between equation S40 and S41 (page S14) there seems to be a half-sentence missing.

We apologize to the reviewer for this. We have updated the missing equation (now S41):

$$p(\alpha|A) = \mathcal{N}\left(\alpha \quad \middle| \quad \frac{s_\alpha^2 A + s_A^2 m_\alpha}{s_A^2 + s_\alpha^2} \quad , \quad \frac{s_A^2 s_\alpha^2}{s_A^2 + s_\alpha^2}\right) \qquad \text{(S41)}$$

13. Page S17 the text says No Feedback Model but Figure S5 says Path Integration Model.

We thank the reviewer for catching this. We have updated the text accordingly

14. Page S18 the text says Cue Combination Model but Figure S6 says Hybrid Model.

We thank the reviewer for catching this. We have updated the text accordingly

15. Figure S13 caption – what is marked in yellow and what is green mean?

We apologize to the reviewer for this. The background color represents which model was the best fit(Fig 13) for that participant: Hybrid model (white), path integration (green), and Cue Combination (yellow). We have updated the caption to include this.
