# Supplementary material for: Combination and competition between path integration and landmark navigation in the estimation of heading direction
Source: PLoS Comput Biol. 2022 Feb 10;18(2):e1009222. doi: 10.1371/journal.pcbi.1009222 (PMC8865642; doi:10.1371/journal.pcbi.1009222)
Supplement: Attachment — Submitted filename: Ekstrom_Competition_PCOMPBIOL-D-21-01111_R1_review.pdf [file pcbi.1009222.s020.pdf]

Review of PCOMPBIOL-D-21-01111-R1, Harootonian, Ekstrom, & Wilson
"Combination and competition between path integration and landmark navigation in the estimation of heading direction"

The authors have, for the most part, satisfactorily addressed my previous comments, and I think the manuscript is in fine shape. However, I do have a couple of lingering questions based on their responses.

**R1.2:** Sorry, I misspoke in my previous comment. What troubled me was that the original manuscript said, "Participants appeared to switch from *cue combination* to cue competition as the offset grew larger," whereas Zhao & Warren (2015) had observed a switch from *visual landmarks* to path integration, a pattern consistent with cue competition.

The authors have now changed that line to, "Participants appeared to switch from *visual cue integration* to path integration as the offset grew larger." But what is "visual cue integration"? This is the only place they use that phrase.

I agree with the larger point that this pattern is consistent with the Hybrid model with a Kalman gain = 1.

**R1.5:** The authors now seem to say that "a sampling interpretation of the Hybrid model" is "similar to Robust cue integration" (Knill, 2007), (Line 510ff). If this is the case, can they articulate what the Hybrid model contributes over and above robust cue integration?

**R2.1:** Note that there is a standard interpretation of ΔBIC values, which is exactly what the authors plot in Fig. 10A (see Raferty, 1995). ΔBIC > 6 indicates "strong" and >10 indicates "very strong" evidence, so the authors could address the reviewer's comment by adding such a vertical dotted line in Fig. 10A.
